# Myocardial effects of angiotensin II compared to norepinephrine in an animal model of septic shock

**DOI:** 10.1186/s13054-022-04161-3

**Published:** 2022-09-18

**Authors:** Bruno Garcia, Fuhong Su, Laurence Dewachter, Raphaël Favory, Amina Khaldi, Alexander Moiroux-Sahraoui, Filippo Annoni, Francisco Vasques-Nóvoa, Estela Rocha-Oliveira, Roberto Roncon-Albuquerque, Geraldine Hubesch, Hassane Njimi, Jean-Louis Vincent, Fabio S. Taccone, Jacques Creteur, Antoine Herpain

**Affiliations:** 1 Experimental Laboratory of Intensive Care, Université Libre de Bruxelles, Brussels, Belgium; 2 Department of Intensive Care, Centre Hospitalier Universitaire de Lille, Lille, France; 3 Laboratory of Physiology and Pharmacology, Université Libre de Bruxelles, Brussels, Belgium; 4 Department of Intensive Care, Erasme University Hospital, Université Libre de Bruxelles, Brussels, Belgium; 5 Cardiovascular R&D Center, Faculty of Medicine, University of Porto, Porto, Portugal; 6 Department of Emergency and Intensive Care Medicine, São João Hospital Center, Porto, Portugal

**Keywords:** Septic shock, Septic cardiomyopathy, Vasopressors, Inflammation, Renin-angiotensin system

## Abstract

**Background:**

Angiotensin II is one of the vasopressors available for use in septic shock. However, its effects on the septic myocardium remain unclear. The aim of the study was to compare the effects of angiotensin II and norepinephrine on cardiac function and myocardial oxygen consumption, inflammation and injury in experimental septic shock.

**Methods:**

This randomized, open-label, controlled study was performed in 20 anesthetized and mechanically ventilated pigs. Septic shock was induced by fecal peritonitis in 16 animals, and four pigs served as shams. Resuscitation with fluids, antimicrobial therapy and abdominal drainage was initiated one hour after the onset of septic shock. Septic pigs were randomly allocated to receive one of the two drugs to maintain mean arterial pressure between 65 and 75 mmHg for 8 h.

**Results:**

There were no differences in MAP, cardiac output, heart rate, fluid balance or tissue perfusion indices in the two treatment groups but myocardial oxygen consumption was greater in the norepinephrine-treated animals. Myocardial mRNA expression of interleukin-6, interleukin-6 receptor, interleukin-1 alpha, and interleukin-1 beta was higher in the norepinephrine than in the angiotensin II group.

**Conclusions:**

In septic shock, angiotensin II administration is associated with a similar level of cardiovascular resuscitation and less myocardial oxygen consumption, and inflammation compared to norepinephrine.

**Supplementary Information:**

The online version contains supplementary material available at 10.1186/s13054-022-04161-3.

## Background

Septic shock remains a major problem in the intensive care unit, with an estimated frequency of 10.4% [1]. Vasopressor therapy is a cornerstone of the complex medical management of patients with septic shock. Norepinephrine (NE) is the first-line vasopressor choice in these patients [2], increasing vascular tone by stimulating *α*-adrenergic receptors and myocardial contractility by stimulating *β*-adrenergic receptors [3]. Adrenergic agents have various non-hemodynamic effects, including increased glycolysis and altered immune responses [4]. NE can dysregulate the immune response by attenuating the production of pro-inflammatory mediators, such as interleukin (IL)-6 or tumor necrosis factor (TNF)-*α*, and increasing anti-inflammatory IL-10 production [5]. Adrenergic receptor stimulation can increase myocardial oxygen consumption, downregulate *β*-adrenergic receptors and reverse adrenergic G protein coupling, resulting in an inhibitory response to catecholamines and impaired myocardial contractility, especially in case of prolonged administration [6–8]. These potential deleterious effects have led to the search for non-catecholaminergic drugs to reduce exposure to catecholamines [2, 9–11].

In the ATHOS III trial, adding synthetic angiotensin II (Ang II) to NE during septic shock resuscitation was associated with significantly increased arterial blood pressure compared to placebo [12]. Ang II administration was also associated with an increased probability of survival in a subgroup of patients with catecholamine-resistant vasodilatory shock and high renin levels at baseline [13].

Ang II has been implicated in the pathophysiology of chronic cardiac disease with pro-inflammatory effects mediated by the angiotensin II receptor 1 (AT_1_R) [14–16]. The safety profile of Ang II therapy in sepsis is not fully defined, but it may increase systemic and myocardial inflammation and cardiomyocyte apoptosis, which are implicated in the pathophysiology of septic cardiomyopathy [17–19].

Since the effects of Ang II on septic cardiomyopathy, especially pro-inflammatory cardiomyopathy, remain unclear, we used a clinically relevant large animal model of septic shock to investigate the effects of Ang II on cardiac function, myocardial oxygenation, myocardial inflammation, injury and apoptosis.

## Methods

### Study design

The study protocol followed the EU Directive (2010/63/EU) for animal experiments and was approved by the local animal ethics committee (Comité Ethique du Bien-Être Animal; protocol number 724N) from the Université Libre de Bruxelles (ULB) in Brussels (Belgium). Experiments were performed in the Experimental Laboratory of Intensive Care of the ULB (LA1230406) and the ARRIVE guidelines and MQTiPSS recommendations for translational research in sepsis were followed [20, 21].

### Experimental procedure

Animals were randomized in an open-label, controlled study based on an established model of septic shock [22, 23]. Twenty pigs (*Sus scrofa domesticus*, RA-SE Genetics, Belgium) weighing 49 ± 5 kg were randomized to fecal peritonitis (*n* = 16) or sham procedure (*n* = 4, consisting of anesthesia and surgical preparation without sepsis induction). Animals were fasted for 18 h prior to the start of the experiment with free access to water. Thereafter, they were sedated in their enclosure with an intramuscular injection of midazolam (1 mg/kg) and ketamine hydrochloride (20 mg/kg) in the neck. After transportation to the operating room, a peripheral line was placed in a vein of the ear and a 4.5 F arterial catheter (Terumo Medical Company, Belgium) was placed in the left common femoral artery for invasive monitoring of arterial pressure and blood sampling. Following anesthesia induction with an intravenous injection of 3 μg/kg of sufentanyl, 1 mg/kg of propofol and 0.5 mg/kg of rocuronium, endotracheal intubation was performed; general anesthesia was achieved with continuous inhalation of sevoflurane (at 1.8 to 2.5% alveolar concentration) and analgesia with continuous infusion of morphine (0.2–0.5 mg/kg/h, the optimal dose being determined through repeated pain tests, i.e., change in heart rate or blood pressure after nasal septum pinching), in association with rocuronium. Volume-controlled mechanical ventilation (Primus, Draëger, Lübeck, Germany) was applied with a fixed tidal volume of 8 mL/kg, a positive end-expiratory pressure of 5 cmH_2_O, a fraction of inspired oxygen (FiO_2_) adjusted to keep PaO_2_ > 90 mmHg, and a respiratory rate adjusted to maintain an arterial pH between 7.35 and 7.45. For drug infusion, a three-lumen central venous catheter (Terumo Medical Company, Belgium) was inserted percutaneously into the right external jugular vein under ultrasound guidance (Vivid E90, GE Machines, USA).

A pulmonary artery catheter (CCO, Edwards LifeSciences, Irvine, California, USA) was advanced through the left external jugular vein into the pulmonary artery for measurement of right heart pressures and continuous monitoring of cardiac output (CO) and mixed venous oxygen saturation (SvO_2_). The electrocardiogram, intravascular pressures and CO were continuously displayed (SC9000, Siemens, Munich, Germany) and exported to an A/D recording station (Notocord-Hem 4.4, Notocord, France). A pressure sensor catheter (Millar® 5F Pressure Catheter, Texas, USA) was introduced in the right common femoral artery. Pulse pressure variation (PPV) was automatically calculated from the arterial femoral signal using the formula “PPV = PP_max_ – PP_min_ / (PPmax + Ppmin) / 2,” with PP being the pulse pressure (i.e., the difference between systolic and diastolic arterial pressures). A left ventricular (LV) pressure volume catheter (5 Fr, Transonic® Europe BV, Elsloo, The Netherlands) was inserted into the LV through the internal carotid artery and was connected to an ADV500 system (Transonic® Europe BV).

Fluid maintenance was achieved using a balanced crystalloid solution (Plasmalyte, Baxter, USA) at a perfusion rate of 5 to 10 mL/kg/h, aiming to maintain the PPV < 13% [24]. Hypoglycemia was avoided by continuous infusion of a 20% glucose solution (1 to 2 mL/kg/h). A 14 Fr Foley catheter was surgically introduced into the bladder via a supra-pubic mini-laparotomy to monitor urine output and intravesical pressure (IVP). Finally, two abdominal drains were placed on each side of the abdominal cavity for the later introduction of autologous feces.

**Fig. 1:**
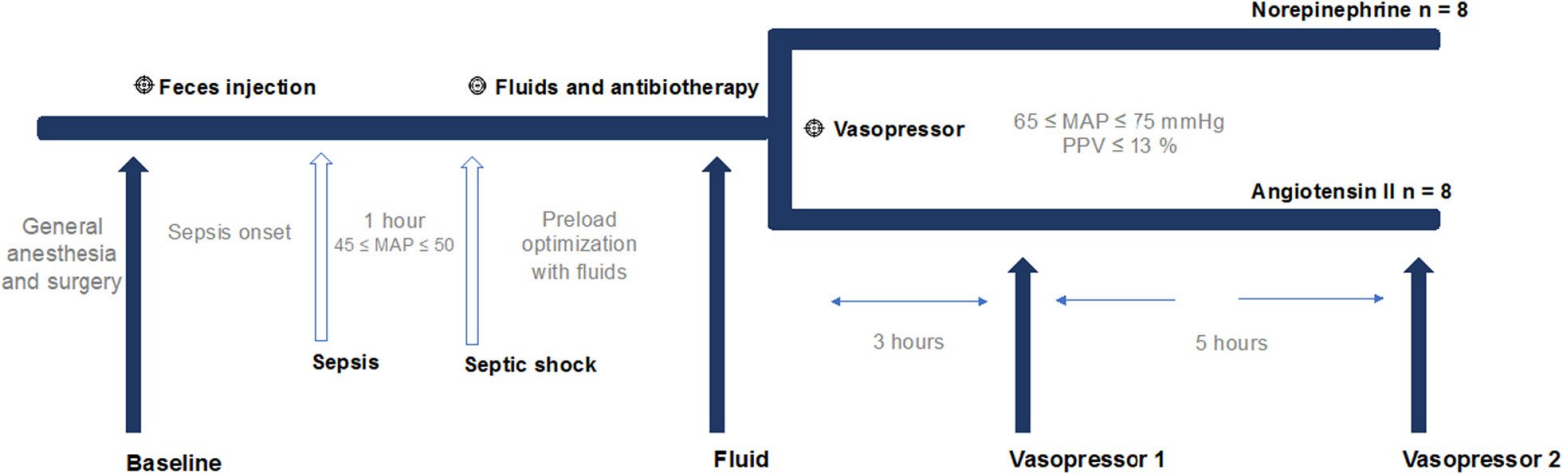
Protocol timeline. At the end of the instrumentation and after hemodynamic stabilization, baseline measurements were obtained. Animals were allowed to develop sepsis until a severe hypotensive state arbitrarily set at a mean arterial pressure (MAP) ≤ 50 mmHg (corresponding to the sepsis time-point) was reached. Severe hypotension (between 45 and 50 mmHg MAP) was left untreated for one hour. The end of this period was defined as the septic shock time-point. Thereafter, full fluid resuscitation was started. Achievement of this objective was called the fluid time-point. According to prior randomization, a continuous infusion of NE or Ang II was then added in addition to the resuscitation fluids. The final two time-points were defined as vasopressor 1 (VP1) after 3 h of vasopressor administration and vasopressor 2 (VP2) after 8 h of vasopressor administration

The experimental protocol and study time-points are shown in Fig. 1. Briefly, the *baseline* time-point was considered as the moment 2 h after the end of the instrumentation, when hemodynamic stabilization had been achieved. Sepsis was then induced by an intraperitoneal instillation of 3 g/kg of autologous feces, previously collected from the animal’s enclosure and diluted in 300 mL of 5% glucose solution. The maintenance infusion rate was reduced to 1 mL/kg/h until the animal developed severe hypotension, arbitrarily defined as a mean arterial pressure (MAP) between 45 and 50 mmHg (corresponding to the *sepsis* time-point). Severe hypotension was left untreated for one hour, and the end of this period was defined as the *septic shock* time-point. Fluid resuscitation was then started with 10 mL/kg/h of balanced crystalloid and 10 mL/kg/h of colloid (Geloplasma, Fresenius Kabi, France), with the objective to restore the PPV to < 13% or MAP ≥ 65 mmHg. Achievement of this objective was considered as the fluids time-point. At this point, the peritoneal drains were opened to remove peritoneal liquid and antimicrobial therapy was started, consisting of an intravenous administration of 2 g of amoxicillin-clavulanic acid every 8 h.

At this stage, animals were randomly allocated to administration of NE or Ang II (Sigma-Aldrich, St. Louis, USA); continuous infusion of both drugs was titrated to achieve a MAP of 65–75 mmHg. The last two, v*asopressor,* time-points (VP1 and VP2) were reached after 3 and 8 h of vasopressor administration, respectively.

The abdominal wall was opened surgically (without opening the peritoneum) in all the animals when the IVP increased to ≥ 12 mmHg, to limit an excessive increase in intra-abdominal pressure (IAP), which could have resulted in abdominal compartment syndrome [25].

After completion of the experiment, the animals were euthanized with 40 mL of 7.5% potassium chloride injection under deep anesthesia. Autopsies were then rapidly performed. Myocardial samples from the free LV wall were stored in an RNA later solution (Invitrogen™, *RNAlater*™ Stabilization Solution, ThermoFisher Scientific, MA, USA) for biological evaluation, flash frozen in liquid nitrogen, or embedded in paraffin after overnight fixation in formaldehyde for immunohistochemistry.

Triple product, calculated as heart rate*maximal ventricular systolic pressure*d*P*/d*t*_max_ (beats/mmHg^2^/s^2^ * 10^5^) was used as a surrogate of cardiac work and myocardial oxygen consumption [26, 27].

LV pressure–volume (PV) loop assessment (as illustrated in Additional file 1: Figure S1) and derived indices, blood sample handling, biomarker quantification, and molecular biology assays (including evaluation of myocardial apoptosis and mRNA and protein expression levels) are described in the supplemental digital content (Additional file 1: Table S1).

### Statistical analysis

All analyses were predefined. All data are presented as mean ± sd (standard deviation) or median [25–75%] unless otherwise stated. To take into account the repeated measurements structure of the data, linear mixed-effects polynomial regression models with restricted maximum likelihood estimation (REML) and first-order autoregressive covariance structure (AR1) were used to examine the differences in all analyzed variables among the groups at the different considered time-points. The group and the time-point were considered as fixed effects in the fitted model. Interaction effects between groups and time were also tested. Post hoc multiple comparison procedure using Tukey HSD test was considered. This test allows for all possible pairwise comparisons while keeping the family-wise error low. Model checking was performed by inspection of residual and normal plots. When the normality of the residuals was rejected, the analyzed variable was log-transformed to fit the normality requirement of the mixed model. Multiple imputation was used to impute missing values. All statistical tests were two-tailed, and a *p* < 0.05 was considered statistically significant. Data were analyzed using Prism (GraphPad Software Inc., USA) and R software (R Foundation for Statistical Computing, Vienna, Austria).

## Results

### Septic shock induction

**Fig. 2:**
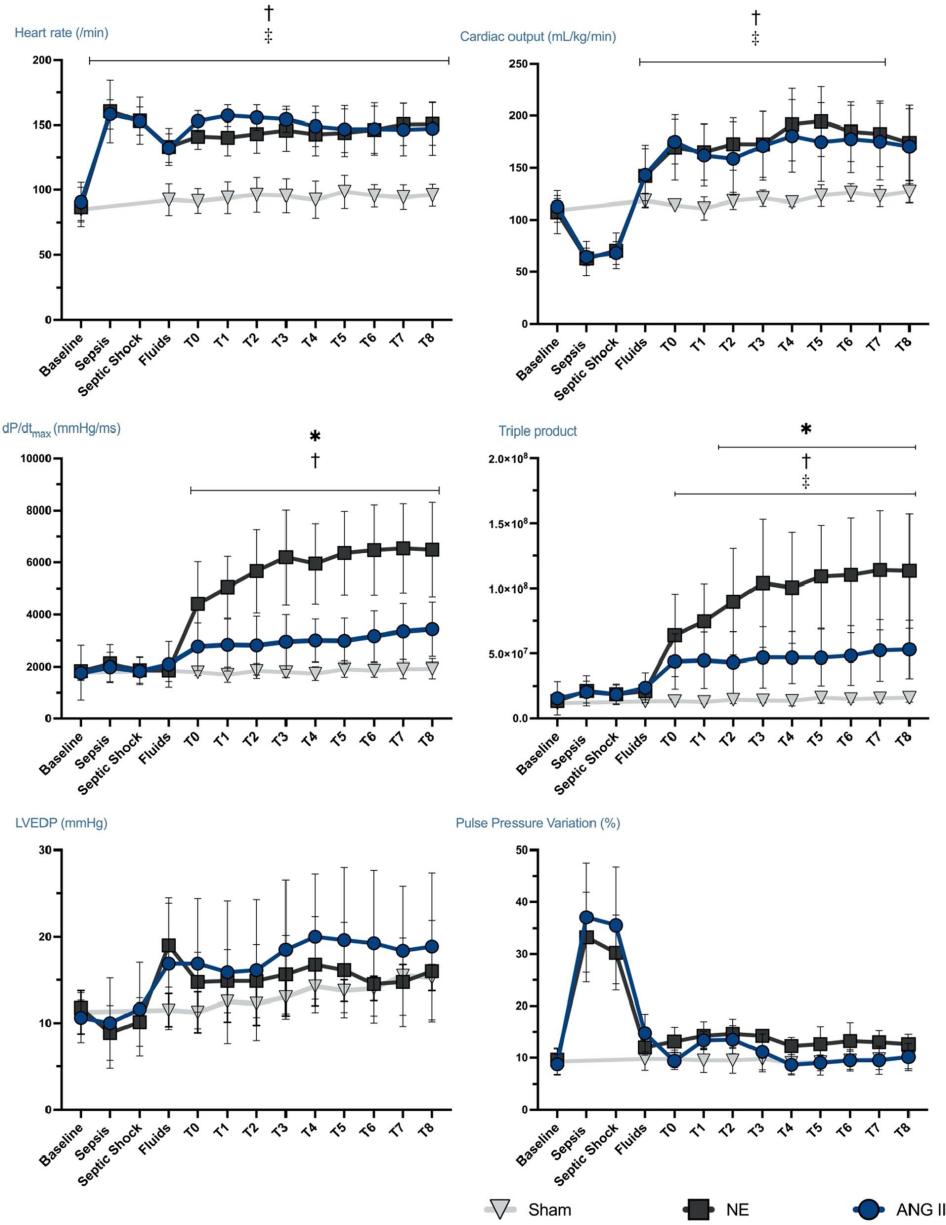
Hemodynamic variables before and during vasopressor exposure in the three groups. Cardiac output and heart rate were not statistically significantly different in the two intervention groups. Norepinephrine (NE) was associated with a higher left ventricular (LV) dP/dTmax and higher triple product (heart rate*ventricular systolic pressure*dP/dT_max_), surrogate of myocardial consumption, than angiotensin (Ang) II. This difference persisted during the 8 h of vasopressor administration. LV end diastolic volume (LVEDV) increased during fluid resuscitation and pulse pressure variation was maintained at <13% throughout the vasopressor administration T0 to T8 correspond to the eight hours of vasopressor exposure, the time-point “vasopressor 1” correspond to T3; time-point “vasopressor 2” correspond to T8. NE: black lines (*n* = 8); Ang II: blue lines (*n* = 8); Sham: white lines (*n* = 4). Values are expressed as mean ± standard deviation. *p value < 0.05 between NE and ANG II. ^†^*p* value < 0.05 between NE and Sham. ^‡^*p* value < 0.05 between ANG II and Sham. *p* value < 0.05 compared to baseline for NE (^§^), Ang II (^ll^) and Sham (**) groups

All 16 animals in the intervention groups developed severe hypotension and tachycardia, associated with decreased SvO_2_ and increased veno-arterial CO_2_ partial pressure difference (PCO_2_ gap) compared to baseline (Tables 1 and 2). The mean time to reach the *Sepsis* time-point was similar in the two treatment groups (5.9 ± 1.4 h for NE vs. 5.7 ± 1.4 h for Ang II, *p* = 0.97). There were no statistically significant differences in hemodynamic, respiratory or biological variables between the treatment groups until the administration of the vasopressor (Fig. 2, Tables 1 and 2, Additional file 1: Tables S4, S5, and S6). The mean dose of NE required to maintain the MAP between 65 and 75 mmHg was 0.58 ± 0.40 microg/kg/min at VP1 and 0.80 ± 0.52 microg/kg/min at VP2. The mean Ang II dose was 261 ± 125 ng/kg/min at VP1 and 1051 ± 775 ng/kg/min at VP2. One animal developed atrial fibrillation in the Ang II group. The total cumulative fluid balance between baseline and the end of the experiment was 161 ± 30 mL/kg with NE and 150 ± 39 mL/kg with Ang II. The experiment lasted 17.7 ± 1.6 h in the NE group and 17.7 ± 1.3 h in the Ang II group (since BL to the euthanasia).

**Table 1:** Hemodynamic variables in the three groups at the different study time-points

Variables		Baseline	Septic	Fluids	Vasopressor 1	Vasopressor 2
Mean ± SD			Shock			
HR (/min)	NE	87 ± 15	153 ± 18	133 ± 14	146 ± 16	151 ± 16
	Ang II	91 ± 15	153 ± 11	132 ± 11	154 ± 10	147 ± 20
CO (mL/min/kg)	NE	107 ± 21	70 ± 17	142 ± 29	173 ± 32	174 ± 36
	Ang II	113 ± 11	68 ± 11	143 ± 23	171 ± 33	170 ± 36
SV (mL/kg)	NE	1.2 ± 0.2	0.4 ± 0.1	1.1 ± 0.2	1.2 ± 0.2	1.2 ± 0.2
	Ang II	1.2 ± 0.1	0.4 ± 0.1	1.0 ± 0.1	1.1 ± 0.2	1.2 ± 0.2
MAP (mmHg)	NE	74 ± 7	49 ± 3	58 ± 5	68 ± 3	68 ± 3
	Ang II	76 ± 8	49 ± 2	55 ± 4	68 ± 2	69 ± 4
LVEDV (mL)	NE	155 ± 45		143 ± 40	124 ± 31	132 ± 37
	Ang II	151 ± 50		154 ± 17	127 ± 31	152 ± 40
LVESV (mL)	NE	90 ± 28		70 ± 20	56 ± 18	62 ± 14
	Ang II	91 ± 42		93 ± 19	75 ± 25	80 ± 29
LVEDP (mmHg)	NE	12 ± 1		19 ± 5	16 ± 4	16 ± 6
	Ang II	11 ± 3		17 ± 8	18 ± 8	19 ± 8
Tau_Log_ (ms)	NE	20 ± 3		13 ± 4	11 ± 2	12 ± 6
	Ang II	19 ± 3		15 ± 8	12 ± 2	12 ± 6
V30 (mL)	NE	193 ± 56		153 ± 27	143 ± 40	146 ± 28
	Ang II	191 ± 59		172 ± 23	152 ± 35	164 ± 29
Chamber stiffness constant *β* (milliliters^−1^)	NE	0.04 ± 0.02		0.07 ± 0.03	0.06 ± 0.02	0.07 ± 0.03
	Ang II	0.04 ± 0.02		0.06 ± 0.03	0.05 ± 0.03	0.04 ± 0.02
d*P*/d*T*_max_ (mmHg/s)	NE	1838 ± 246	1870 ± 246	1860 ± 427	6198 ± 1827*	6491 ± 1809*
	Ang II	1766 ± 1050	1832 ± 512	2088 ± 875	2953 ± 1044	3437 ± 1044
d*P*/d*T*_max_/EDV ratio	NE	10.2 ± 4.8		12.4 ± 5.6	35.4 ± 8.6*	36.7 ± 12*
	Ang II	12.7 ± 8.3		8.9 ± 2.4	20.1 ± 7.7*	17.3 ± 3.74*
PRSW (mmHg)	NE	57 ± 20		57 ± 17	101 ± 21*	98 ± 38
	Ang II	48 ± 20		63 ± 15	85 ± 13	78 ± 12
*E*_max_ (mmHg/mL)	NE	0.7 ± 0.3		0.9 ± 0.3	1.6 ± 0.7	1.4 ± 0.9
	Ang II	0.8 ± 0.3		0.9 ± 0.2	1.2 ± 0.4	0.8 ± 0.2
V0 (mL)	NE	− 37 ± 16		− 25 ± 23	− 36 ± 26	− 30 ± 40
	Ang I	− 27 ± 27		− 31 ± 52	− 37 ± 36	− 55 ± 37
V100 (mL)	NE	104 ± 26		87 ± 14	49 ± 19	51 ± 11
	Ang II	96 ± 41		108 ± 11	83 ± 40	65 ± 23
*E*_a_ (mmHg/mL)	NE	1.5 ± 0.2		1.6 ± 0.3	1.8 ± 0.8	2.1 ± 0.8
	Ang II	1.4 ± 0.3		1.5 ± 0.2	1.8 ± 0.4	1.6 ± 0.3
*E*_a_/*E*_max_ ratio	NE	1.8 ± 1		1.7 ± 0.7	1.3 ± 0.6	1.6 ± 0.3
	Ang II	1.5 ± 0.5		2.2 ± 1.1	1.7 ± 0.6	2.0 ± 0.5
EF (%)	NE	40 ± 13		39 ± 10	49 ± 17	47 ± 8
	Ang II	46 ± 16		35 ± 7	47 ± 10	44 ± 10
RAP (mmHg)	NE	11 ± 1	10 ± 1	13 ± 2	13 ± 3	14 ± 3
	Ang II Sham	9 ± 2	11 ± 2	12	12 ± 2	12 ± 2
mPAP (mmHg)	NE	22 ± 2	25 ± 4	29 ± 5	29 ± 5	29 ± 5
	Ang II	24 ± 4	26 ± 5	28 ± 3	29 ± 3	29 ± 3

PV loop analysis was obtained at baseline, fluids, vasopressor 1 and vasopressor 2

HR, heart rate; MAP, mean arterial pressure; SV, stroke volume; CO, cardiac output; RAP, right atrial pressure; LVEDV, left ventricular end diastolic volume; LVESV, left ventricular end systolic volume; LVEDP, left ventricular end diastolic pressure; EF, ejection fraction; PRSW, preload recruitable stroke work; *E*_max_, left ventricular maximal elastance; *E*_a_, effective arterial elastance; *E*_a_/*E*_max_, left ventriculo-arterial coupling; V30, LV volume at 30 mmHg on the End Diastolic Pressure Volume Relationship; V0, LV volume at 0 mmHg on the End Systolic Pressure Volume Relationship; V100, LV volume at 100 mmHg on the End Systolic Pressure Volume Relationship; NE, norepinephrine; Ang, angiotensin

*p value < 0.05 between NE and Ang II

**Table 2:** Biological and oxygenation values in the three groups at the different study time-points

Variables		Baseline	Septic	Fluids	Vasopressor 1	Vasopressor 2
Mean ± SD			Shock			
SVO_2_ (%)	NE	66 ± 4	48 ± 5	70 ± 7	76 ± 5	72 ± 6
	Ang II	61 ± 4	49 ± 7	70 ± 5	71 ± 7	72 ± 7
Lactate (mmol/L)	NE	1 ± 0.1	1.5 ± 0.7	1.6 ± 0.6	1.9 ± 0.6	1.9 ± 1.1
	Ang II	0.9	1.3 ± 0.2	1.6 ± 0.2	1.3 ± 0.2	1.9 ± 0.6
PCO_2_ gap (mmHg)	NE	8 ± 4	15 ± 5	5 ± 3	5 ± 1	5 ± 5
	Ang II	8 ± 4	15 ± 5	5 ± 5	5 ± 3	5 ± 4
BE (mmol/L)	NE	8.8 ± 2	4.9 ± 3.5	7.7 ± 3.4	7.3 ± 2.9	6.1 ± 2.9
	Ang II	8.6 ± 1.6	5.5 ± 1.8	7.6 ± 2.2	7.9 ± 2.2	7.9 ± 2.7
Creatinine (mg/dL)	NE	1 ± 0.1		1.9 ± 0.3	1.8 ± 0.4	1.7 ± 0.6
	Ang II	1 ± 0.2		1.9 ± 0.3	1.9 ± 0.5	1.8 ± 0.4
Albumin (g/L)	NE	25 ± 3		15 ± 2	10 ± 3	9 ± 2
	Ang II	24 ± 5		15 ± 3	11 ± 2	10 ± 3
Hematocrit (%)	NE	25.6 ± 1.9	42.3 ± 2.7	23.8 ± 2.9	28.4 ± 2.7	27.5 ± 3.6
	Ang II	25.7 ± 2.7	41.5 ± 2.9	24.4 ± 3	26.3 ± 4	25.1 ± 4.1
Troponin I (ng/mL)	NE	0.3 ± 0.3				2.3 ± 2
	Ang II	0.4 ± 0.4				1 ± 0.6
TNF-*α* (pg/mL)	NE	118 [106–156]		210 [181–238]	164 [89–144]	144 [131–172]
Median IQR	Ang II	146 [130–156]		246 [162–278]	165 [137–200]	151 [138–196]
IL-6 (pg/mL)	NE	13 [12, 13]		1446 [990–1621]	1197 [566–2271]	750 [428–1208]
Median IQR	Ang II	12 [11, 12]		1435 [1170–1513]	793 [402–2017]	473 [287–889]
IL-10 (pg/mL)	NE	10 [9–11]		19 [17–22]	19 [17–20]	16 [15–20]
Median IQR	Ang II	8 [8, 9]		20 [15–23]	22 [21–27]	20 [19–23]

CO_2_ gap, veno-arterial difference in CO_2_ partial pressure; SVO_2_, mixed venous oxygen saturation; BE, base excess; IL, interleukin; TNF, tumor necrosis factor; NE, norepinephrine; Ang, angiotensin

### Global hemodynamics and tissue perfusion indices

After fluid resuscitation and during vasopressor therapy, MAP was maintained between 65 and 75 mmHg in all treated animals with a mean value of 69 ± 3 mmHg during the eight-hour exposure. Fluid administration and vasopressor therapy restored SvO_2_ and CO_2_ gap to within normal values in the two groups (Table 2). There were no statistically significant differences in CO, HR, or stroke volume (SV) over time in the two intervention groups (Fig. 2 and Table 1).

LV contractility, assessed by the d*P*/d*T*_max_, increased in both groups with the vasopressor infusion, and this increase persisted in both groups using the d*P*/d*t*_max_/EDV ratio as a preload-independent contractility index (Table 1). d*P*/d*t*_max_ was higher with NE than with Ang II throughout the 8 h of vasopressor administration (Fig. 2). The LV PRSW and *E*_max_ increased under NE exposure (Table 1). The theoretical volume extrapolated from the ESVPR at 100 mmHg of LV pressure (V100) deceased under norepinephrine treatment, corresponding to an increase in contractility (Table 1).

Estimation of the myocardial volume oxygen consumption (MVO_2_), assessed using the “Triple product,” a surrogate for MVO_2_ taking into account heart rate, ventricular pressure maximal range, and ventricular contractility (via LV d*P*/d*t*_max_), was larger with NE than with Ang II (Fig. 2).

There were no significant differences between the two groups in LV maximal elastance, arterial elastance, preload recruitable stroke work (PRSW), or other PV loop-derived indices (Table 1).

Time comparisons and comparison with the Sham group are shown in Additional file 1: Table S2–S3.

### Evaluation of inflammation, myocardial mRNA expression, myocardial injury, and apoptosis

**Fig. 3:**
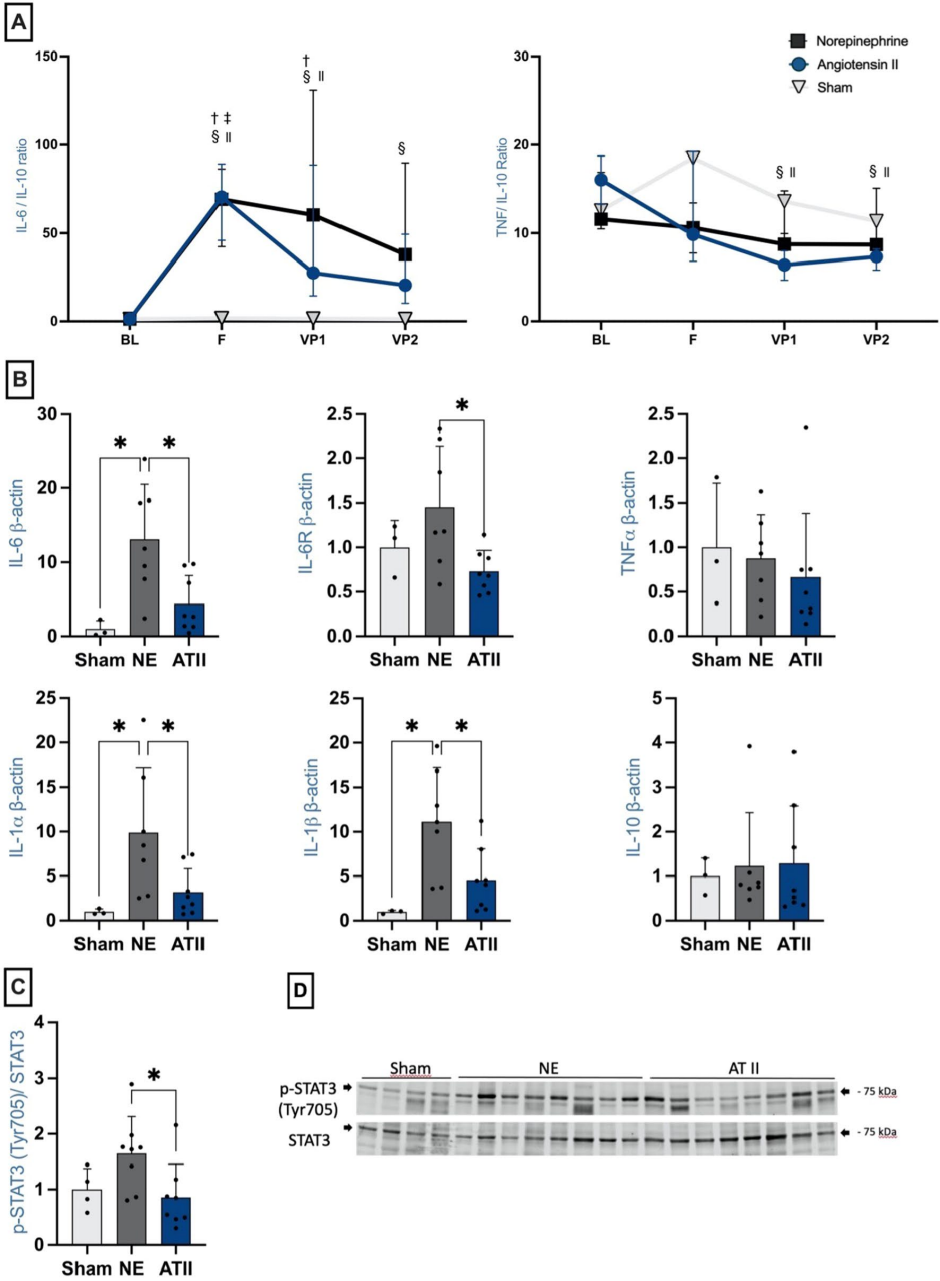
Left ventricular inflammatory markers. **A**: Ratios between plasma interleukin (IL)-6 and IL-10 and between tumor necrosis factor (TNF)-*α* and IL-10. ^†^*p* value < 0.05 between NE and Sham. ^‡^*p* value < 0.05 between Ang II and Sham. *p* value < 0.05 compared to baseline for NE (^§^), Ang II (^ll^) and Sham (**) groups. **B**: LV relative mRNA expression of IL-6, IL-6 receptor (IL-6R), IL-1*α* and -1*β* Relative quantification was achieved using the comparative 2^−ΔΔCt^ method by normalization with the housekeeping gene (ActB-actin). Results are expressed as relative fold increase above the mean value of LV relative mRNA expression of the sham group arbitrarily fixed at 1. *p < 0.05. NE: black boxes (*n* = 7); ATII: blue boxes (*n* = 8); sham: white boxes (*n* = 3). **C**: Myocardial LV STAT3 activation (assessed as Tyr705 phosphorylation normalized to total STAT3 expression). Results are expressed as relative fold increase above the mean value of LV relative mRNA expression of the sham group arbitrarily fixed at 1. **D:** pSTAT3 / STAT3 gels. Uncropped gels are available in the Additional file 2. *p < 0.05. NE: black boxes (*n* = 8); ATII: blue boxes (*n* = 8); sham: white boxes (*n* = 4)

At the *Fluids* time-point, circulating levels of pro-inflammatory cytokines (TNF-*α* and IL-6) had increased similarly in the two treatment groups in response to sepsis (Table 2); Anti-inflammatory IL-10 levels were increased in the Ang II group at VP1 compared to baseline. Circulating IL-6 levels remained high in the NE group at VP1 and VP2, but decreased at VP2 in the Ang II group. Circulating levels of TNF-*α* were not statistically significantly different from baseline values at VP1 and VP2 (Table 2). IL-6/IL-10 and TNF-*α*/IL-10 ratios at the different time-points are shown in Fig. 3.

LV pro-inflammatory cytokines (expressed as mRNA expression of IL-6 and its receptor (IL-6R), IL1-*α*, and IL1-*β*) were upregulated in the NE compared to the Ang II group (Fig. 3). There were no statistically significant differences in mRNA expressions of TNF-*α* and IL-10 (Fig. 3). As shown in Additional file 1: Figure S2, mRNA expressions of intercellular adhesion molecule-1 (ICAM-1) and vascular adhesion molecule-1 (VCAM-1) were higher in the NE than in the Ang II group. There were no statistically significant differences in ICAM-2 expression between the groups.

The activation of signal transducer and activator of transcription 3 (STAT3), the main transcription factor involved in IL-6-mediated signaling, herein evaluated by Tyr705 phosphorylation, were upregulated in NE—compared to Ang II-treated animals (Fig. 3).

Myocardial injury, assessed by high sensitive cardiac troponin I (hs-cTrop) release, was significantly higher at VP2 in animals receiving NE compared to sham animals (*p* = 0.0023), with a similar trend compared to Ang II (*p* = 0.06), (Additional file 1: Figure S3).

The LV pro-apoptotic Bax-to-Bcl2 mRNA expression ratio was higher in the Ang II than in the NE and sham groups (Additional file 1: Figure S2), but the LV apoptotic rate, assessed by TUNEL staining and evaluating the termination of apoptotic processes, was similar in the three groups (Additional file 1: Figure S2).

### Myocardial mRNA and protein expression of adrenergic and angiotensin II receptors

**Fig. 4:**
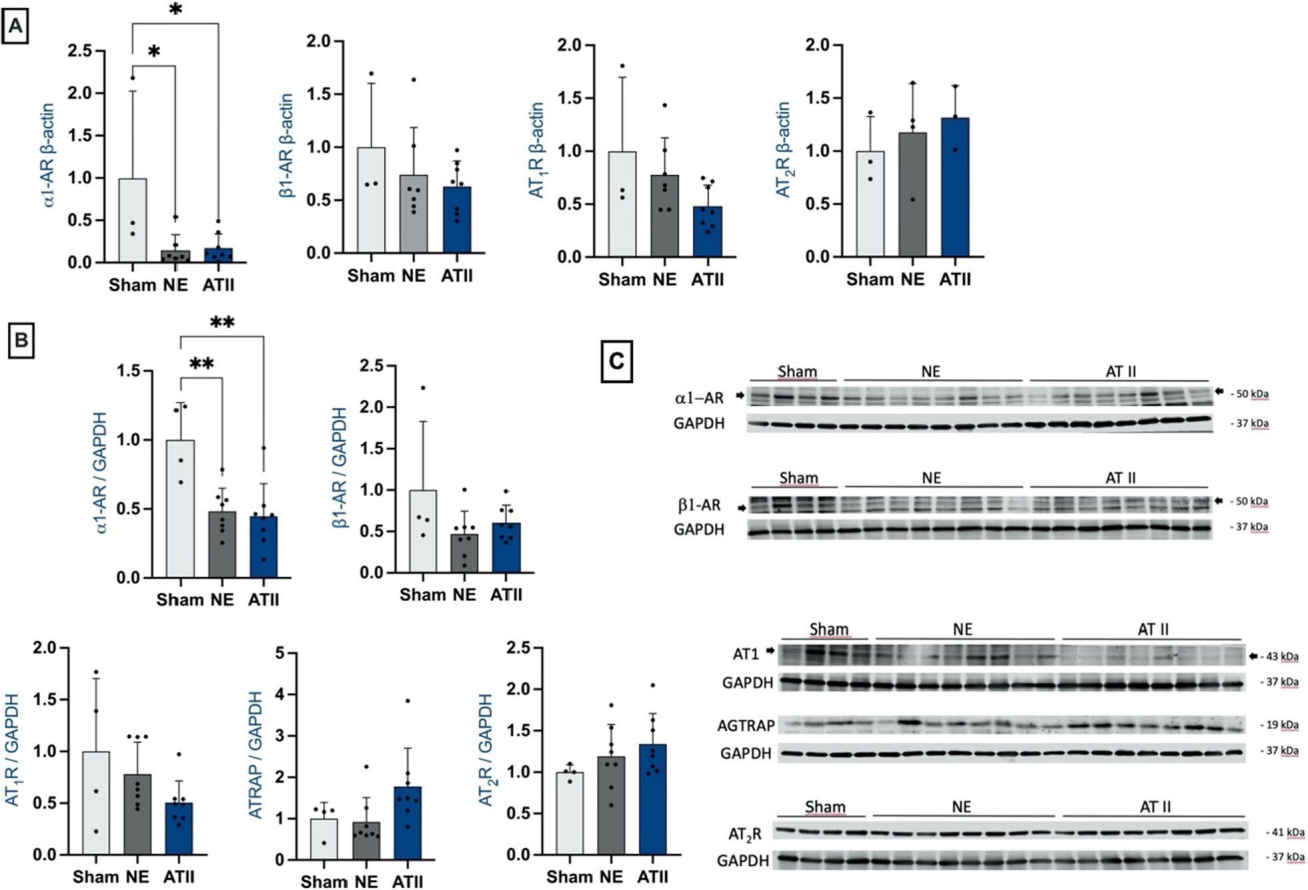
Left ventricular myocardial adrenergic and angiotensin II receptor expression in the three groups. **A:** Left ventricular (LV) relative mRNA expression of angiotensin II receptor type 1 and 2 (AT_1_R and AT_2_R). LV relative mRNA expression of adrenergic receptor alpha 1 (*α*1-AR) and adrenergic receptor beta 1 (*β*1-AR). Relative quantification was achieved using the comparative 2^−ΔΔCt^ method by normalization with the housekeeping gene (ActB). Results are expressed as relative fold increase above the mean value of LV relative mRNA expression of the sham group arbitrarily fixed at 1. *p < 0.05. NE: black boxes (*n* = 7); Ang II: blue boxes (*n* = 8); sham: white boxes (*n* = 3). **B:** Immunoblotting of LV samples of *α*1-AR, *β*1-AR, AT_1_R, and AT_2_R protein expressions. Values are expressed as mean ± standard deviation. **C.** LV adrenoreceptors and angiotensin receptors gels. Uncropped gels are available in the Additional file 2. *p < 0.05. NE: black boxes (*n* = 8); ATII: blue boxes (*n* = 8); sham: white boxes (*n* = 4)

LV mRNA and protein expressions of adrenergic receptor alpha 1 (*α*1-AR) were decreased in the two intervention groups compared to in sham animals, whereas adrenergic receptor beta 1 (*β*1-AR) mRNA and protein expressions were similar (Fig. 4A, B) to those in the sham group. There was no significant difference in Ang II receptor 1 (AT_1_R) or Ang II receptor 2 (AT_2_R) mRNA and protein expression between groups. AT_2_R mRNA and protein expressions were not affected by sepsis or vasopressor choice (Fig. 4), but there was a tendency for AT_1_R mRNA and protein expression to decrease in both intervention groups, mainly in the Ang II group (*p* value = 0.12 and 0.11 for Ang II vs. sham, for mRNA and protein expression, respectively). In addition, protein levels of Type-1 angiotensin II receptor-associated protein (AGTRAP), a negative regulator of the AT_1_R signaling pathway, also tended to be upregulated in the Ang II group (*p* = 0.07 and *p* = 0.21 compared to NE and sham groups, respectively).

## Discussion

In this experimental model of septic shock, Ang II administration combined with optimal fluid administration and antibiotic therapy resulted in a similar degree of cardiovascular resuscitation compared to NE administration in the first hours of septic shock. Load-independent contractility indices derived from PV loop assessment confirmed an intrinsic positive inotropic effect of Ang II in sepsis, with a lower myocardial oxygen consumption than NE. Cardiac inflammation, assessed by myocardial mRNA expression of inflammatory cytokines (IL-6 and its receptor, IL1-*α* and IL1-*β*), was upregulated in the NE compared to the Ang II group and STAT3 signaling was consistent with an IL-6 mediated pathway. AT_2_R mRNA and protein expressions were not affected by sepsis or vasopressor choice.

Administration of Ang II with fluids achieved the same resuscitation objectives and normalization of the tissue perfusion indices SvO_2_ and PCO_2_ gap as did administration of NE. Ang II was associated with a small increase in cardiac contractility, assessed by d*P*/d*T*max, and this increase in contractility was persistent when using d*P*/d*T*max/EDV ratio, a non-preload dependent index [28]. The positive inotropic effect of Ang II has been shown in in vitro studies [29, 30] and one preclinical study in healthy pigs [31]. As expected, cardiac contractility rapidly increased with NE, to a larger extent than in the Ang II group and persisted throughout the study. Nevertheless, MAP, CO and heart rate were similar in the two groups. This observation is consistent with a previous experimental study by Corrêa et al. conducted in a swine model of septic shock, in which there were no significant differences in MAP, CO, or SV between NE and Ang II groups, despite the same fluid resuscitation protocol [32]. These studies highlight the importance of preload optimization with fluids in septic shock, especially when using drugs with a predominantly vasopressor effect [2]; indeed, Wan et al. showed that Ang II infusion alone decreased CO in a non-fluid resuscitated septic shock model [33]. Another potentially beneficial effect of Ang II related to reducing catecholamine exposure is the finding that NE was associated with increased MVO_2_, as assessed using the “Triple product.” This observation suggests higher oxygen consumption with NE administration, with no beneficial effect on tissue perfusion, and may have important clinical implications, especially in patients with ischemic cardiomyopathy or other causes of impaired myocardial perfusion.

Catecholamines are known to have direct pro-inflammatory effects on the myocardium [34], but the observed differences in myocardial inflammation can also be explained by modulation of the renin-angiotensin pathway: Bellomo et al. showed that Ang II infusion reduced renin secretion in septic shock [13] and hypothesized that the decrease in renin secretion could modulate the immune response. The incubation of leukocytes with renin induces the production of pro-inflammatory cytokines, including IL-6 [35], and administration of a renin receptor blocker reduced the pro-inflammatory response and increased survival in a rodent model of sepsis induced by cecal ligation and puncture (CLP) [36].

Another hypothesis that could explain the difference in myocardial inflammation between groups is modulation via angiotensin receptors. Myocardial AT_1_R is downregulated during sepsis [37] and we showed that AT_2_R myocardial mRNA and protein expression were not affected by the vasopressor choice. Moreover, myocardial levels of AGTRAP tended to be upregulated in the Ang II group. We hypothesize that when Ang II is used, the imbalance between myocardial AT_1_R and AT_2_R during sepsis may lead to a predominant AT_2_R-mediated pathway, responsible for an anti-inflammatory effect [38]. This result is in accordance with previous data showing sepsis-induced reduction in the AT_1_R/AT_2_R ratio in human arterial samples exposed to septic stimuli [39]. Finally, it has been shown recently that angiotensin-converting enzyme 2 (ACE2), which transforms Ang II into angiotensin (1–7) (Ang-(1–7)), an anti-inflammatory peptide, was increased during acute respiratory distress syndrome [40] and an increase in Ang-(1–7) concentration was also observed. Hence, we can postulate that a similar decrease in the ACE/ACE2 ratio may occur during sepsis [41, 42], resulting in degradation of Ang II and an anti-inflammatory response mediated by Ang-(1–7), which could be restored by synthetic Ang II perfusion. Moreover, Ang II can also be transformed into alamandine via a double step process through decarboxylases and ACE2, which can also modulate anti-inflammatory properties [43].

These findings, associated with a trend to less myocardial injury as assessed by hs-cTrop release, may be related to an improvement in cardiovascular outcomes after sepsis [44].

The increase in mRNA expression of the Bax/Bcl2 ratio is consistent with the imbalance between AT_1_R/AT_2_R, as AT_2_R stimulation is known to have pro-apoptotic effects [45]. Nevertheless, terminal apoptosis assessed using TUNEL staining was similar between groups, which suggests that the short exposure period was not sufficient to induce apoptosis in this model.

We used a large animal model of peritonitis-induced septic shock that fulfills preclinical research recommendations for clinical relevance and external validity [20, 46].

Dynamic parameters are recommended to assess fluid responsiveness, and here, fluid administration was titrated to maintain a PPV < 13% when hypotension occurred [47]. This was associated with a high fluid balance observed in both groups and explained by the severity of the model. When compared to others studies, fluid administration in swine models is generally high and comparable with our study [48, 49]. The objective of the fluid protocol used here was to optimize preload similarly in both groups, with a PPV < 13% when hypotension occurred, which is not necessarily associated with fluid unresponsiveness [24].The IVP was also monitored and abdominal wall opened to avoid any interaction of increased IAP on hemodynamic management [25]. Instrumentation was performed with a closed chest and pericardium and according to a minimally invasive approach; moreover, as all the catheters were introduced percutaneously under ultrasound guidance, tissue inflammation related to surgery was limited. This model follows other recommendations of the Surviving Sepsis Campaign, including abdominal drainage, adapted antimicrobial therapy, and early vasopressor introduction [2]. We used polymicrobial sepsis as can be observed in humans, which induced severe multiple organ dysfunction. Nevertheless, our study has several limitations: First, due to species differences with humans, normal lactate levels are usually observed in swine septic shock [48–50] despite developing all the criteria for a septic shock and tissular hypoperfusion [51]. Second, the limited number of animals may reduce the probability of showing differences between groups and the open label design could expose a selection bias, but randomization was performed before the start of the experiment and baseline characteristics were well balanced in all groups. Third, the relatively early development of sepsis might explain the absence of significant septic cardiomyopathy and overt molecular changes, limiting our ability to make inferences about the impact of this therapeutic strategy in this situation. However, one could argue that treatment in the early phase of septic shock is of paramount importance in determining the later development of septic cardiomyopathy. In addition, our study was focused on norepinephrine, which has alpha- and beta-adrenergic effects. Here, the study cannot separate these different mechanisms, and a third group with a pure alpha vasopressor, as phenylephrine, would have better clarified the contribution of the different pathways but against SSC recommendations. Finally, the high ang II dose used in our study is similar to that observed in a similar septic shock swine study [32] but higher compared to the average dose used in the ATHOS III study [12]. However, in the ATHOS III study, Ang II was used in association with norepinephrine and the vasoplegia in these patients was lower with a median norepinephrine requirement of 0.34 microg/kg/min at baseline, compared to 0.80 microg/kg/min at VP2 in our experimental model [12]. We investigated the use of Ang II as a single vasopressor agent, instead of in association with NE, but this enabled us to better analyze the specific effects of Ang II.

These results have several implications for vasopressor use in septic shock, especially regarding the safety concerns of Ang II on myocardial inflammation: By reducing renin secretion and stimulating AT_2_R, Ang II could have beneficial effects on local inflammation [13, 38], in addition to the beneficial effects of reducing catecholamine exposure, which can otherwise contribute to impaired cardiac contractility by *β*-adrenergic downregulation [8, 52] and higher MVO_2_.

## Conclusions

In conclusion, in a resuscitated large animal model of septic shock, Ang II administration can restore organ perfusion as efficiently as can NE, resulting in a similar CO, heart rate and MAP, but with less MVO_2_, and inflammation compared to treatment with NE.

## Supplementary Information

**Additional file 1**. **Figure S1.** Pressure-volume loop illustration. **Table S1.** Primers used for real-time quantitative polymerase chain reaction (RTQ-PCR) in porcine myocardial tissue. **Table S2.** Hemodynamic variables in the three groups at the different study time-points. *p-value <0.05 between NE and Ang II. †p-value < 0.05 between NE and Sham. ‡p-value < 0.05 between Ang II and Sham. P-value < 0.05 compared to baseline for NE (§), Ang II (ll) and Sham (**) groups. HR: heart rate; MAP: mean arterial pressure; SV: stroke volume; CO: cardiac output; RAP: right atrial pressure; LVEDV: left ventricular end diastolic volume; LVESV: left ventricular end systolic volume; LVEDP: left ventricularend diastolic pressure; EF: ejection fraction; PRSW: preload recruitable stroke work; Emax: left ventricular maximal elastance; Ea: effective arterial elastance; Ea/Emax: left ventriculo-arterial coupling; V30: LV volume at 30 mmHg on the End Diastolic Pressure Volume Relationship; V100: LV volume at 100 mmHg on the End Systolic Pressure Volume Relationship; NE: norepinephrine; Ang: angiotensin PV loop analysis was obtained at baseline, fluids, vasopressor 1 and vasopressor 2. **Table S3.** Biological and oxygenation values in the three groups at the different study timepoints. *p-value <0.05 between NE and Ang II. †p-value < 0.05 between NE and Sham. ‡p-value < 0.05 between Ang II and Sham. p-value < 0.05 compared to baseline for NE (§), Ang II (ll) and Sham (**) groups. CO2 gap: veno-arterial difference in CO2 partial pressure; SVO2: mixed venous oxygen saturation; BE: base excess; IL: interleukin; TNF: tumor necrosis factor; NE: norepinephrine; Ang: angiotensin. **Table S4.** Respiratory variables. Results are presented as mean + SD. *p-value between NE and Ang II groups. †p-value < 0.05 between NE and Sham groups. ‡p-value < 0.05 between Ang II and Sham groups. p-value < 0.05 compared to baseline for NE (§), Ang II (ll) and Sham (**) groups. PaO2: Arterial partial pressure of oxygen; FiO2 fraction of oxygen inspired; Pplat: plateau pressure; Crs:compliance of the respiratory system; EtCO2: end-tidal carbon dioxide; PaCO2: arterial partial pressure of carbon dioxide. **Table S5.** Blood gas analysis. Values are presented as mean + SD. *p-value between NE and angiotensin II group. †p-value < 0.05 between NE and Sham group. ‡p-value < 0.05 between angiotensin II and Sham group. P-value < 0.05 compared to baseline for NE (§), angiotensin II (ll) and Sham (**) group. Hb: hemoglobin; Ht: hematocrit. PaO2: arterial partial pressure of oxygen. PaCO2: arterial partial pressure of carbon dioxide. **Table S6.** Biological variables. Values are expressed as mean ± SD. *p-value between NE and Ang II groups. †p-value < 0.05 between NE and Sham groups. ‡p-value < 0.05 between Ang II and Sham groups. p-value < 0.05 compared to baseline for NE (§), Ang II (ll) and Sham (**) groups. ASAT: Aspartate aminotransferase; ALAT Alanine aminotransferase; LDH: Lactate dehydrogenase. **Figure S2.** A. Left ventricular mRNA expression of molecules implicated in Ca^2+^ handling and contractile apparatus [ATPase sarcoplasmic/endoplasmic reticulum Ca^2+^ transporting 2 (SERCA2A) and phospholamban (PLB)] B. LV mRNA expression of the ratio Bax/Bcl2 in norepinephrine (black bars) and angiotensin II groups. *pvalue< 0.05. C. Cardiac apoptotic rate: ratio of apoptotic nuclei (TUNEL-positive or brown nuclei) to total nuclei (brown+blue nuclei) (x100 to be expressed as a percentage). D. mRNA expression of cell adhesion molecules (ICAM1, 2 and VCAM1) and eNOS, iNOS, nNOS in norepinephrine and angiotensin II groups compared to sham group. **Figure S3.** Fold Changes expressed in % between baseline and vasopressor 2 time points

**Additional file 2**. Uncropped gels.

## Data Availability

The datasets used during the current study are available from the corresponding author on reasonable request.
