# Supplementary material for: Myocardial effects of angiotensin II compared to norepinephrine in an animal model of septic shock
Source: Crit Care. 2022 Sep 18;26:281. doi: 10.1186/s13054-022-04161-3 (PMC9482744; doi:10.1186/s13054-022-04161-3)
Supplement: Supplementary file 1 — Additional file 1. Figure S1. Pressure-volume loop illustration. Table S1. Primers used for real-time quantitative polymerase chain reaction (RTQ-PCR) in porcine myocardial tissue. Table S2. Hemodynamic variables in the three groups at the different study time-points. *p-value <0.05 between NE and Ang II. †p-value < 0.05 between NE and Sham. ‡p-value < 0.05 between Ang II and Sham. P-value < 0.05 compared to baseline for NE (§), Ang II (ll) and Sham (**) groups. HR: heart rate; MAP: mean arterial pressure; SV: stroke volume; CO: cardiac output; RAP: right atrial pressure; LVEDV: left ventricular end diastolic volume; LVESV: left ventricular end systolic volume; LVEDP: left ventricularend diastolic pressure; EF: ejection fraction; PRSW: preload recruitable stroke work; Emax: left ventricular maximal elastance; Ea: effective arterial elastance; Ea/Emax: left ventriculo-arterial coupling; V30: LV volume at 30 mmHg on the End Diastolic Pressure Volume Relationship; V100: LV volume at 100 mmHg on the End Systolic Pressure Volume Relationship; NE: norepinephrine; Ang: angiotensin PV loop analysis was obtained at baseline, fluids, vasopressor 1 and vasopressor 2. Table S3. Biological and oxygenation values in the three groups at the different study timepoints. *p-value <0.05 between NE and Ang II. †p-value < 0.05 between NE and Sham. ‡p-value < 0.05 between Ang II and Sham. p-value < 0.05 compared to baseline for NE (§), Ang II (ll) and Sham (**) groups. CO2 gap: veno-arterial difference in CO2 partial pressure; SVO2: mixed venous oxygen saturation; BE: base excess; IL: interleukin; TNF: tumor necrosis factor; NE: norepinephrine; Ang: angiotensin. Table S4. Respiratory variables. Results are presented as mean + SD. *p-value between NE and Ang II groups. †p-value < 0.05 between NE and Sham groups. ‡p-value < 0.05 between Ang II and Sham groups. p-value < 0.05 compared to baseline for NE (§), Ang II (ll) and Sham (**) groups. PaO2: Arterial partial pressure of oxygen; FiO2 f [file 13054_2022_4161_MOESM1_ESM.docx]

**SUPPLEMENTAL DIGITAL CONTENT**

**MYOCARDIAL EFFECTS OF ANGIOTENSIN II COMPARED TO NOREPINEPHRINE IN AN ANIMAL MODEL OF SEPTIC SHOCK**

Bruno Garcia^1,2^; Fuhong Su^1^; Laurence Dewachter^3^; Raphaël Favory^2^; Amina Khaldi^1,4^; Alexander Moiroux-Sahraoui^1^; Filippo Annoni^1,4^; Francisco Vasques-Nóvoa^5^, Estela Rocha-Oliveira^5^, Roberto Roncon-Albuquerque Jr^5,6^, Geraldine Hubesch^3^; Hassane Njimi^4^; Jean-Louis Vincent^1,4^, Fabio S Taccone^1,4^; Jacques Creteur^1,4^; Antoine Herpain^1,4^

^1^Experimental Laboratory of Intensive Care, Université Libre de Bruxelles, Brussels, Belgium

^2^Department of Intensive care, Centre Hospitalier Universitaire de Lille, Lille, France

^3^Laboratory of Physiology and Pharmacology, Université Libre de Bruxelles, Brussels, Belgium

^4^Department of Intensive Care, Erasme University Hospital, Université Libre de Bruxelles, Brussels, Belgium

^5^Cardiovascular R&D Center, Faculty of Medicine, University of Porto, Porto, Portugal

^6^Department of Emergency and Intensive Care Medicine, São João Hospital Center, Porto, Portugal

**METHODS:**

**Triple product**

Triple product, calculated as heart rate*maximal ventricular systolic pressure*dP/dt_max_ (beats/mmHg^2^/s^2^*10^5^) was used as a surrogate of myocardial oxygen consumption^1^.

**Left ventricular pressure-volume loop processing**

Pressure-volume loops (PVLS) were obtained from a 5 Fr pressure-volume catheter (5F VSL P/V catheter, Transonic Europe, Netherlands) inserted in the left ventricle through the right carotid artery and connected to an ADV500 device (Transonic Europe, Netherlands). The optimal catheter positioning in the middle of the left ventricular (LV) cavity and with the tip at the LV apex was confirmed under echocardiographic and PVL shape guidance. Pressure and volume signals were zeroed to atmospheric pressure and calibrated with the stroke volume (SV) from the pulmonary artery catheter monitoring, and were exported to an A/D recording station (Notocord-Hem 4.4, Notocord, France).

At the four main time-points (baseline, fluids, vasopressor 1, and vasopressor 2), under brief apnea, preload was reduced by progressive inferior vena cava (IVC) occlusion using an intravascular balloon inflation. The exact timing of the end of systole was determined by the maximal ventricular elastance computation, using the iterative method. This enabled measurement of the end-systolic pressure-volume relationship slope along its linear portion, which represented the maximal LV elastance (E_max_). LV end-diastolic volume (EDV), end-systolic volume (ESV), end-diastolic pressure (EDP), stroke work (SW), Tau logistic, stroke volume (SV), and effective arterial elastance (E_a_, i.e., the ratio between ESP and SV) were also recorded. The preload reduction was also used to determine the preload recruitable stroke work (PRSW), as the slope of the linear correlation between each EDV and its related SW for this heartbeat. Finally, ventricular-arterial coupling was computed as the ratio between E_a_ and E_max_. All measurements were replicated three times under brief apnea and then averaged ^2^. Maximal pressure rises over time (dP/dtmax) and maximal pressure falls over time (dP/dtmin) were recorded hourly during vasopressor therapy.

The End Systolic Pressure Volume Relationship (ESPVR) was computed, from which the LV volume at 0 mmHg (V0) and at 100 mmHg (V100) of filling pressure were extrapolated^2,3^.

For the LV diastolic function assessment, the exponential curve of the End Diastolic Pressure Volume Relationship (EDPVR) was computed, from which we extracted the LV volume theoretically reached at 30 mmHg of filling pressure (V30)^3^ and the *Chamber stiffness constant β*^2^.

**Figure S1: Pressure-volume loop illustration**

**Blood sampling and blood gas and biochemical analyses**

Arterial and venous blood gas measurements were obtained from femoral and pulmonary artery blood samples using a blood gas and electrolyte analyzer (Cobas b-123, Roche, Switzerland) at all time-points (baseline, sepsis, septic shock, fluids, vasopressor 1, and vasopressor 2; see Figure 1). Systemic plasma samples for biochemical analysis were collected from the femoral artery at the four main time points (baseline, fluids, vasopressor 1, and vasopressor 2) and immediately centrifuged and frozen for further processing in a dedicated veterinary lab (Synlab veterinary, Brussels, Belgium).

**Enzyme-linked immunosorbent assay (ELISA)**

Circulating plasma levels of interleukin (IL)-6, IL-10, and tumor necrosis factor (TNF)-α were determined with Quantikine Porcine IL-6, IL-10 and TNF-a ELISA kits (R&D system, Minneapolis, USA), respectively, according to the manufacturer’s protocols.

**Immunohistochemistry - Terminal Deoxynucleotidyl Transferase dUTP Nick-End Labeling (TUNEL) Staining**

Detection of cardiac cells in apoptosis was achieved using Terminal Deoxynucleotidyl Transferase dUTP Nick-End Labeling (TUNEL) staining using the ApopTag® Plus Peroxidase In Situ Apoptosis Detection Kit (Sigma-Aldrich, USA) according to the manufacturer’s instructions. Porcine lung samples from previous experiments were used as positive controls. For each cardiac specimen, twenty randomly chosen x 40 fields were examined. The cardiac apoptotic rate was calculated as the ratio of apoptotic nuclei (TUNEL-positive or brown nuclei) to total nuclei (brown+blue nuclei) (x100 to be expressed as percentages). All counts were performed by two independent investigators in a blinded fashion. The mean value was used for analysis.

**Real-time quantitative polymerase chain reaction (RTq-PCR)**

Total RNA was extracted from snap-frozen LV tissue stored at -80°C in RNA later solution (Invitrogen™, *RNA later*™ Stabilization Solution, ThermoFisher Scientific, MA, USA), using the RNeasy Mini kit (QIAGEN, Germany). RNA concentration was determined by a standard spectrophotometric technique, using a Nanodrop® ND-1000 (Isogen Life Science, Netherlands). RNA integrity was assessed by visual inspection of GelRed (Biotium, California) -stained agarose gels. Reverse transcription was performed using random hexamer primers and Superscript II Reverse Transcriptase (Invitrogen, Carlsbad, USA), according to the manufacturer’s instructions. For RTq-PCR, sense and anti-sense primers were designed using the Primer3 program for *sus scrofa* Bax, Bcl-2, sarcoplasmic reticulum calcium transport ATP-ase pump 2 (SERCA2), phospholamban (PLB), ryanodine receptor 2 (RYR2), IL-1β, IL-1α, IL-6, IL-6 receptor (IL-6R), IL-10, IL 10-receptor (IL-10R), TNF-α, intercellular adhesion molecule 1 (ICAM1) and 2 (ICAM2), vascular cell adhesion molecule 1 (VCAM1), endothelial (NOS3 or eNOS), inducible (NOS2 or iNOS) and neuronal (NOS1 or nNOS) NO-synthases, beta1- (ADRB1), beta2-(ADRB2) and beta3-(ADRB3) adrenergic receptors, and alpha1A- adrenergic receptor (ADRA1A) (m) RNA sequences (Table S1). To avoid inappropriate amplification of residual genomic DNA, intron-spanning primers were selected when exon sequences were known. For each sample, the ampliﬁcation reaction was performed in triplicate using SYBRGreen PCR Master Mix (Quanta Biosciences, Gaithersburg, MD), speciﬁc primers, and diluted template complementary DNA using an iCycler system (BioRad Laboratories). Relative quantification was achieved using the comparative 2^-ΔΔCt^ method by normalization with the housekeeping gene (beta-actin). Results are expressed as relative fold increase above the mean value of LV relative mRNA expression of the sham group arbitrarily ﬁxed at 1.

**Table S1** Primers used for real-time quantitative polymerase chain reaction (RTQ-PCR) in porcine myocardial tissue.

| Genes | Primer Sequences |
| --- | --- |
| **Alpha1A- adrenergic receptor (ADRA1A)**  Sense  Antisense | 5'- AGTGATGCCCATTGGGTCTTT -3'  5'- ATGGGGTTGATGCAGCTGTT -3' |
| **B-cell Lymphoma-2 (Bcl-2)**  Sense  Antisense | 5’- GACTTTGCCGAGATGTCCAG -3’  5’- ACAATCCTCCCCCAGTTCA -3’ |
| **Bcl-2 associated X apoptosis regulator (Bax)**  Sense  Antisense | 5’- CGCATTGGAGATGAACTGG -3’  5’- CGCCACTCGGAAAAAGACT -3’ |
| **Beta-actin**  Sense  Antisense | 5’- CAGCAGATGTGGATCAGCAA -3’  5’- CAAGTCCGCCTAGAAGCATT -3’ |
| **Beta1-adrenergic receptor (**β1-AR**)**  Sense  Antisense | 5'- ACCCCAAGTGCTGCGATTT -3'  5'- ATGCACAAGGGCACGTAGAA -3' |
| **Beta2-adrenergic receptor (**β2-AR**)**  Sense  Antisense | 5'- GATTCACAGGGGAGGAACTGTAG -3'  5'- TTGTTTAGTGTTTGGCTGGGAG -3' |
| **Beta3-adrenergic receptor (**β3-AR**)**  Sense  Antisense | 5'- CAGAATGAGCCCTGTGGAGAT -3'  5'- AGGTTGGTGAAAAGCCACTTG -3' |
| **Intercellular adhesion molecule 1 (ICAM1)**  Sense  Antisense | 5’- ATTGTGAGGGGTGTCGAAGT -3’  5’- TTCCCAGTTGTGTGTTTCCA -3’ |
| **Intercellular adhesion molecule 2 (ICAM2)**  Sense  Antisense | 5’- GGGCTCAGTGGAAGCTGTAT -3’  5’- GGGAGAACACGCTGATGTTG -3’ |
| **Interleukin-1alpha**  Sense  Antisense | 5'- GTCCAAAACGAAGACGAACC -3'  5'- CCATATTGCCATGCTTTTCC -3' |
| **Interleukin-1beta**  Sense  Antisense | 5'- CACCCAAAACCTGGACCTT -3'  5'- TGCCTGATGCTCTTGTTC -3' |
| **Interleukin-6 (IL-6)**  Sense  Antisense | 5'- CCACCAGGAACGAAAGAGAG -3'  5'- AGTAGCCATCACCAGAAGCAG -3' |
| **Interleukin-6 receptor (IL-6R)**  Sense  Antisense | 5'- CCGGAGGGAGACAACTCTTT -3'  5'- GGCTGCAAGATTCCATAACC -3' |
| **Interleukin-10 (IL-10)**  Sense  Antisense | 5'- TCATCAATTTCTGCCCTGTG -3'  5'- TGTAGACACCCCTCTCTTGGA -3' |
| **Interleukin-10 receptor (IL-10R)**  Sense  Antisense | 5'- TTCAAGTCCGAGCGTTTCTT-3'  5'- GGTTTCGTCATTGGTCGTCT -3' |
| **Nitric oxide synthase 1 (NOS1 or nNOS)**  Sense  Antisense | 5'- CTTCAATCTCTTTGTTCACCTCCTC -3'  5'- GAGTATTACTCGTCAATTAAAAGAT -3' |
| **Nitric oxide synthase 2 (NOS2 or iNOS)**  Sense  Antisense | 5'- CTGCATGGATAAGTACAGGCTGACC-3'  5'- AGCTTCTGATCAATGTCATGAGCAA -3' |
| **Nitric oxide synthase (NOS3 or eNOS)**  Sense  Antisense | 5'- CTTTCCTGTTGGCCTGACCA -3'  5'- CCGGTTACTCAGACCCAAGG -3' |
| **Phospholamban**  Sense  Antisense | 5'- AAACAGCCAAGGCTGCCTAAA -3'  5'- GATACCAGGAAGGCAGGAAGC -3' |
| **Ryanodin receptor 2 (RYR2)**  Sense  Antisense | 5'- GTGAAGCAGCCCAAGGGTAT -3'  5'- AAGGGACAGTGAGGCATTCG -3' |
| **Sarcoplasmic/endoplasmic reticulum Ca2+ transporting ATPase 2 (SERCA2)**  Sense  Antisense | 5'- GGGAAAACCTTGCTGGAACT -3'  5'- CTTCGCCTTCTTCAAACCAA -3' |
| **Tumor necrosis factor-alpha (TNF-a)**  Sense  Antisense | 5'- TCTGGACTTTGCTGAATCTGG -3'  5'- GGTTTCGTCATTGGTCGTCT -3' |
| **Vascular cell adhesion molecule 1 (VCAM1)**  Sense  Antisense | 5’- GGAATTTACGTGTGCGAGGG-3’  5’- TCCCTGGGAGCAACTTGAAC -3’ |

**Immunoblotting**

Immunoblotting was performed as previously described^4^. Briefly, 40 μg of LV protein were separated by SDS-PAGE using 4-20% gradient polyacrylamide gels (Criterion™ TGX™ Precast Gels, #5671095, Bio-Rad) and then electroblotted into nitrocellulose membranes (Trans-Blot® Turbo, Bio-Rad). Blots were blocked and incubated with primary antibodies to angiotensin II type 1 receptors (AT_1_R; Santa Cruz Biotechnology, sc-1173), type-1 angiotensin II receptor-associated protein (AGTRAP, ThermoFisher, PA5-88400/ IRDye 800) and type 2 angiotensin II receptors (AT_2_R; Abcam, ab92445), adrenoceptors alpha 1A (α1-AR; Abcam, ab137123) and beta 1 (β1-AR; Abcam, ab3442), signal transducer and activator of transcription 3 (STAT3; Cell Signaling, #9139) and phospho-STAT3 (Tyr705; Cell Signaling, #9145) overnight at 4ºC. The immunoblots were subsequently washed and incubated with 700 nm or 800 nm infra-red dye-conjugated antibodies (LI-COR Biosciences; #926-68020 and #926-32211). The membrane was imaged by scanning at 800 and 700 nm with an Odyssey Infrared Imaging System (LICOR Biosciences). GAPDH was used as an internal control (Abcam, ab8245) and the control group was set as reference.

**RESULTS**

**Table S2: Hemodynamic variables in the three groups at the different study time-points**

| VARIABLES  Mean +/- SD |  | Baseline | Septic Shock | Fluids | Vasopressor 1 | Vasopressor 2 |
| --- | --- | --- | --- | --- | --- | --- |
| HR (/min) | NE  Ang II  Sham | 87 ± 15  91 ± 15  85 ± 8 | 153 ± 18^§^  153 ± 11^ll^ | 133 ± 14^†,§^  132 ± 11^‡,ll^  92 ± 12 | 146 ± 16^†,§^  154 ± 10^‡,ll^  96 ± 13 | 151 ± 16^†,§^  147 ± 20^‡,ll^  96 ± 8 |
| CO  (ml/min/kg) | NE  Ang II  Sham | 107 ± 21  113 ± 11  109 ± 11 | 70 ± 17^§^  68 ± 11^ll^ | 142 ± 29^†,§^  143 ± 23^‡^  119 ± 7 | 173± 32^†,§^  171 ± 33^‡,ll^  121 ± 8 | 174 ± 36^†,§^  170± 36^‡,ll^  127 ± 10 |
| SV  (ml/kg) | NE  Ang II  Sham | 1.2 ± 0.2  1.2 ± 0.1  1.3 ± 0.1 | 0.4 ± 0.1^§^  0.4 ± 0.1^##^ | 1.1 ± 0.2  1.0 ± 0.1  1.2 ± 0.1 | 1.2 ± 0.2  1.1 ± 0.2  1.2 ± 0.1 | 1.2 ± 0.2  1.2 ± 0.2  1.2 ± 0.1 |
| MAP  (mmHg) | NE  Ang II  Sham | 74 ± 7^†^  76 ± 8^‡^  67 ± 3 | 49 ± 3  49 ± 2 | 58 ± 5^†§^  55 ± 4^‡,ll^  67 ± 3 | 68 ± 3^§^  68 ± 2^ll^  68 ± 5 | 68 ± 3^§^  69 ± 4^ll^  68 ± 1 |
| LVEDV  (mL) | NE  Ang II  Sham | 155 ± 45  151 ± 50  144 ± 27 |  | 143 ± 40  154 ± 17  144 ± 27 | 124 ± 31  127 ± 31  138 ± 15 | 132 ± 37  152 ± 40  133 ± 16 |
| LVESV  (mL) | NE  Ang II  Sham | 90 ± 28  91 ± 42  67 ± 5 |  | 70 ± 20  93± 19  83 ± 17 | 56 ± 18  75 ± 25  102 ± 12 | 62 ± 14  80 ± 29  95 ± 13 |
| LVEDP  (mmHg) | NE  Ang II  Sham | 12± 1  11 ± 3  11 ± 2 |  | 19 ± 5^§^  17 ± 8^ll^  11 ± 2 | 16 ± 4^§^  18 ± 8^ll^  13 ± 2 | 16 ± 6  19 ± 8^ll^  15 ± 2 |
| Tau _Log_  (ms) | NE  Ang II  Sham | 20 ± 3  19 ± 3  19 ± 2 |  | 13 ± 4^§^  15 ± 8  14 ± 1 | 11 ± 2^§^  12 ± 2^ll^  16 ± 3 | 12 ± 6^§^  12 ± 6  15 ± 3 |
| V30  (mL) | NE  Ang II  Sham | 193 ± 56  191 ± 59  211 ± 23 |  | 153 ± 27^†^  172 ± 23  204 ± 18 | 143 ± 40^†^  152 ± 35^‡^  201 ± 5 | 146 ± 28^†^  164 ± 29^‡^  196 ± 7 |
| Chamber stiffness constant β (milliliters^-1^) | NE  Ang II  Sham | 0.04 ± 0.02  0.04 ± 0.02  0.03 ± 0.02 |  | 0.07 ± 0.03  0.06 ± 0.03  0.03. ± 0.02 | 0.06 ± 0.02  0.05 ± 0.03  0.02 ± 0.01 | 0.07 ± 0.03  0.04 ± 0.02  0.03 ± 0.03 |
|  |  |  |  |  |  |  |
| dP/dT_max_  (mmHg/s) | NE  Ang II  Sham | 1838 ± 246  1766 ± 1050  1781 ± 300 | 1870±246  1832±512 | 1860 ± 427  2088 ± 875  1848 ± 202 | 6198 ± 1827^*,†,§^  2953 ± 1044^*^  1799 ± 208 | 6491 ± 1809^*,†,§^  3437 ± 1044^*,ll^  1920 ± 383 |
| dP/dT_max_ / EDV ratio | NE  Ang II  Sham | 10.2 ± 4.8  12.7 ± 8.3  12.1 ± 1.2 |  | 12.4 ± 5.6  8.9 ± 2.4  10.9 ± 1.5 | 35.4 ± 8.6^*,†,§^  20.1 ± 7.7^*^  8.8 ± 2.6 | 36.7 ± 12^*,†,§^  17.3 ± 3.74^*^  10.6 ± 4.8 |
| PRSW  (mmHg) | NE  Ang II  Sham | 57 ± 20  48 ± 20  51 ± 7 |  | 57 ± 17  63 ± 15  62 ± 13 | 101 ± 21^†,§^  85 ± 13^‡^  46 ± 3 | 98 ± 38^†,§^  78 ± 12  53 ± 14 |
| E_max_  (mmHg/mL) | NE  Ang II  Sham | 0.7 ± 0.3  0.8 ± 0.3  0.8 ± 0.1 |  | 0.9 ± 0.3  0.9 ± 0.2  0.8 ± 0.1 | 1.6 ± 0.7^§^  1.2 ± 0.4  0.6 ± 0.1 | 1.4 ± 0.9^§^  0.8 ± 0.2  0.8 ± 0.3 |
| V0  (mL) | NE  Ang II  Sham | -37 ± 16  -27 ± 27  -35 ± 8 |  | -25 ± 23  -31 ± 52  -40 ± 6 | -36 ± 26  -37 ± 36  -51 ± 17 | -30 ± 40  -55 ± 37  -50 ± 19 |
| V100  (mL) | NE  Ang II  Sham | 104 ± 26  96 ± 41  89 ± 9 |  | 87 ± 14  108 ± 11  104 ± 20 | 49 ± 19^†,§^  83 ± 40  127 ± 10 | 51 ± 11^†,§^  65 ± 23  118± 12 |
| E_a_  (mmHg/mL) | NE  Ang II  Sham | 1.5 ± 0.2  1.4 ± 0.3  1.3 ± 0.3 |  | 1.6 ± 0.3  1.5 ± 0.2  1.4 ± 0.2 | 1.8 ± 0.8  1.8 ± 0.4  1.5 ± 0.3 | 2.1 ± 0.8^§^  1.6 ± 0.3  1.3 ± 0.1 |
| E_a_/E_max_ ratio | NE  Ang II  Sham | 1.8 ± 1  1.5 ± 0.5  1.6 ± 0.2 |  | 1.7 ± 0.7  2.2 ± 1.1  1.9 ± 0.1 | 1.3 ± 0.6  1.7 ± 0.6  2.6 ± 0.9 | 1.6 ± 0.3  2.0 ± 0.5  1.9 ± 0.9 |
| EF  (%) | NE  Ang II  Sham | 40 ± 13  46 ± 16  44 ± 5 |  | 39 ± 10  35 ± 7  41 ± 3 | 49 ± 17  47 ± 10  36 ± 18 | 47 ± 8  44 ± 10  42 ± 16 |
| RAP  (mmHg) | NE  Ang II  Sham | 11 ± 1  9 ±2  8 ± 3 | 10 ± 1  11 ± 2 | 13 ± 2  12  9 ± 3 | 13 ± 3  12 ± 2^ll^  10 ± 1 | 14 ± 3  12 ± 2^ll^  12 ± 1^**^ |
| mPAP  (mmHg) | NE  Ang II  Sham | 22 ± 2  24 ± 4  21 ± 3 | 25 ± 4  26 ± 5 | 29 ± 5^§^  28 ± 3  22 ± 2 | 29 ± 5^†,§^  29 ± 3^ll^  25 ± 2 | 29 ± 5^†,§^  29 ± 3^‡,ll^  22 ± 1 |

*p-value <0.05 between NE and Ang II. ^†^p-value < 0.05 between NE and Sham. ^‡^p-value < 0.05 between Ang II and Sham. P-value < 0.05 compared to baseline for NE (^§^), Ang II (^ll^) and Sham (**) groups.

HR: heart rate; MAP: mean arterial pressure; SV: stroke volume; CO: cardiac output; RAP: right atrial pressure; LVEDV: left ventricular end diastolic volume; LVESV: left ventricular end systolic volume; LVEDP: left ventricular end diastolic pressure; EF: ejection fraction; PRSW: preload recruitable stroke work; E_max_: left ventricular maximal elastance; E_a_: effective arterial elastance; E_a_/E_max_: left ventriculo-arterial coupling; V30: LV volume at 30 mmHg on the End Diastolic Pressure Volume Relationship; V100: LV volume at 100 mmHg on the End Systolic Pressure Volume Relationship; NE: norepinephrine; Ang: angiotensin

PV loop analysis was obtained at baseline, fluids, vasopressor 1 and vasopressor 2.

**Table S3: Biological and oxygenation values in the three groups at the different study timepoints.**

| VARIABLES  Mean +/- SD |  | Baseline | Septic Shock | | Fluids | | Vasopressor 1 | | Vasopressor 2 | |  |
| --- | --- | --- | --- | --- | --- | --- | --- | --- | --- | --- | --- |
| SVO_2_  (%) | *NE*  *Ang II*  *Sham* | 66 ± 4  61 ± 4  67 ± 5. | | 48 ± 5^§^  49 ± 7^ll^ | | 70 ± 7  70 ± 5^ll^  64 ± 4 | | 76 ± 5 ^†,§^  71 ± 7^ll^  63 ± 3 | | 72 ± 6^†^  72 ± 7^ll^  63 ± 2 | |
| Lactate  (mmol/L) | *NE*  *Ang II*  *Sham* | 1 ± 0.1  0.9  0.9 | | 1.5 ± 0.7  1.3 ± 0.2 | | 1.6 ± 0.6  1.6 ± 0.2  0.9 | | 1.9 ± 0.6^†^  1.3 ± 0.2  0.9 | | 1.9 ± 1.1^†^  1.9 ± 0.6  0.9 | |
| PCO_2_ gap  (mmHg) | *NE*  *Ang II*  *Sham* | 8 ± 4  8 ± 4  5 ± 3 | | 15 ± 5^§^  15 ± 5^ll^ | | 5 ± 3  5 ± 5  7 ± 1 | | 5 ± 1  5 ± 3  1 ± 2 | | 5 ± 5  5 ± 4  6 ± 2 | |
| BE  (mmol/L) | *NE*  *Ang II*  *Sham* | 8.8 ± 2  8.6 ± 1.6  8.6 ± 2.3 | | 4.9 ± 3.5^§^  5.5 ± 1.8^ll^ | | 7.7 ± 3.4  7.6 ± 2.2^‡^  11.2 ± 1.5 | | 7.3 ± 2.9  7.9 ± 2.2  10.6 ± 1.7 | | 6.1 ± 2.9^†,§^  7.9 ± 2.7^‡^  12.1 ± 1.2 | |
| Creatinine  (mg/dL) | *NE*  *Ang II*  *Sham* | 1 ± 0.1  1 ± 0.2  0.9 ± 0.2 | |  | | 1.9 ± 0.3^†,§^  1.9 ± 0.3^‡,ll^  0.1 ± 0.1 | | 1.8 ± 0.4^†,§^  1.9 ± 0.5^‡,ll^  0.9 ± 0.1 | | 1.7 ± 0.6^§^  1.8 ± 0.4^ll^  1 ± 0.1 | |
| Albumin  (g/L) | *NE*  *Ang II*  *Sham* | 25 ± 3  24 ± 5  27 ± 2 | |  | | 15 ± 2^†,§^  15 ± 3^‡,ll^  25 ± 1 | | 10 ± 3^†,§^  11 ± 2^‡,ll^  25 ± 3 | | 9 ± 2^†,§^  10 ± 3^‡,ll^  24 ± 2 | |
| Hematocrit  (%) | *NE*  *Ang II*  *Sham* | 25.6 ± 1.9  25.7 ± 2.7  27.6 ± 1.6 | | 42.3 ± 2.7^§^  41.5 ± 2.9^ll^ | | 23.8 ± 2.9  24.4 ± 3  28.9 ± 1.5 | | 28.4 ± 2.7  26.3 ± 4  28.3 ± 1.13 | | 27.5 ± 3.6  25.1 ± 4.1  27.2 ± 1.1 | |
| Troponin I  (ng/mL) | *NE*  *Ang II*  *Sham* | 0.3 ± 0.3  0.4 ± 0.4  0.3 ± 0.2 | |  | |  | |  | | 2.3 ± 2^†,§^  1 ± 0.6  0.3 ± 0.2 | |
| TNF-α  (pg/mL)  Median IQR | *NE*  *Ang II*  *Sham* | 118 [106-156]  146[130-156]  158 [149-167] | |  | | 210[181-238] ^§^  246 [162-278]^ll^  168 [143-193] | | 164 [144-89]  165 [137-200]  149 [124-165] | | 144 [131-172]  151 [138-196]  143 [112-144] | |
| IL-6  (pg/mL)  Median IQR | *NE*  *Ang II*  *Sham* | 13 [12-13]  12[11-12]  16 [15-16] | |  | | 1446 [990-1621]^§^  1435[1170-1513]^ll^  19 [17-20] | | 1197[566-2271]^†,§^  793 [402-2017]^‡,ll^  17 [16-23] | | 750 [428-1208]^§^  473 [287-889]  16 [14-20] | |
| IL-10  (pg/mL)  Median IQR | *NE*  *Ang II*  *Sham* | 10 [9-11]  8 [8-9]  11 [10-12] | |  | | 19 [17-22]  20 [15-23]  12 [11-12] | | 19 [17-20]  22 [21-27]^‡^  12[11-12] | | 16 [15-20]  20 [19-23]  12 [10-12] | |

*p-value <0.05 between NE and Ang II. ^†^p-value < 0.05 between NE and Sham. ^‡^p-value < 0.05 between Ang II and Sham. p-value < 0.05 compared to baseline for NE (^§^), Ang II (^ll^) and Sham (**) groups.

CO_2_ gap: veno-arterial difference in CO_2_ partial pressure; SVO_2_: mixed venous oxygen saturation; BE: base excess; IL: interleukin; TNF: tumor necrosis factor; NE: norepinephrine; Ang: angiotensin.

As illustrated in Figure S2, there were no changes in the ratio between SERCA2 and other calcium cycling related players, including PLB or RYR2. Because nitric oxide modulates cardiac function, through key regulation of calcium channels implicated in excitation-contraction coupling and β-adrenergic signaling, myocardial expression of nitric oxide synthases (eNOS, iNOS and nNOS) was evaluated but there was no significant change in mRNA expression (Figure S2).

**Table S4**: Respiratory variables

| VARIABLES  Mean +/- SD |  | Baseline | Septic Shock | Fluids | Vasopressor 1 | | Vasopressor 2 |
| --- | --- | --- | --- | --- | --- | --- | --- |
| PaO_2_/FiO_2_  (mmHg) | NE  Ang II  Sham | 320 ± 48  321 ± 31  345 ± 27 | 262 ± 35  254 ± 34 | 274 ± 81  287 ± 51  316 ± 29 | | 260 ± 43  269 ± 78  340 ± 55 | 263 ± 33  292 ± 103  328 ± 47 |
| Pplat  (mmHg) | NE  Ang II  Sham | 19 ± 3  17 ± 2  18 ± 4 | 20 ± 2  19 ± 2 | 21 ± 4  20 ± 2  21 ± 2 | | 21 ± 4  22 ± 3^ll^  19 ± 2 | 22 ± 3^§,†^  22 ± 3^ll,‡^  17 ± 3 |
| Crs  (mL/cmH_2_O) | NE  Ang II  Sham | 31 ± 6  33 ± 5  33 ± 10 | 28 ± 7  29 ± 4  18 ± 4 | 27 ± 7  28 ± 6  25 ± 3 | | 27 ± 6  29 ± 4^ll^  28 ± 3 | 25 ± 6^§,†^  24 ± 5^‡,ll^  33 ± 6 |
| EtCO_2_  (mmHg) | NE  Ang II  Sham | 41 ± 3  44 ± 3  43 ± 4 | 49 ±4^§^  53 ± 5^ll^ | 46 ± 8  51 ± 5  46 ± 2 | | 44 ± 8  50 ± 5  46 ± 3 | 47 ± 9  46 ± 5  48 ± 3 |
| PaCO_2_  (mmHg) | NE  Ang II  Sham | 44 ± 2  47 ± 4  46 ± 3 | 52 ± 6^§^  54 ± 4^ll^ | 50 ± 8  57 ± 7^ll^  52 ± 2 | | 51 ± 8^§^  55 ± 3^ll^  51 ± 1 | 48 ± 5^§^  52 ± 4^ll^  51 ± 1 |

Results are presented as mean + SD. *p-value between NE and Ang II groups. ^†^p-value < 0.05 between NE and Sham groups. ^‡^p-value < 0.05 between Ang II and Sham groups. p-value < 0.05 compared to baseline for NE (^§^), Ang II (^ll^) and Sham (**) groups.

PaO_2_: Arterial partial pressure of oxygen; FiO_2_ fraction of oxygen inspired; Pplat: plateau pressure; Crs: compliance of the respiratory system; EtCO2: end-tidal carbon dioxide; PaCO2: arterial partial pressure of carbon dioxide.

**Table S5:** **Blood gas analysis.** Values are presented as mean + SD. *p-value between NE and angiotensin II group. ^†^p-value < 0.05 between NE and Sham group. ^‡^p-value < 0.05 between angiotensin II and Sham group. P-value < 0.05 compared to baseline for NE (^§^), angiotensin II (^ll^) and Sham (**) group. Hb: hemoglobin; Ht: hematocrit. PaO_2_: arterial partial pressure of oxygen. PaCO_2_: arterial partial pressure of carbon dioxide.

| VARIABLES  Mean +/- SD |  | Baseline | Septic Shock | Fluids | Vasopressor 1 | | Vasopressor 2 | |
| --- | --- | --- | --- | --- | --- | --- | --- | --- |
| pH | NE  Ang II  Sham | 7.49 ± 0.02  7.47 ± 0.04  7.47 ± 0.01 | 7.39 ± 0.07^§^  7.39 ± 0.03^ll^ | 7.43 ± 0.01  7.39 ± 0.05^‡^  7.46 ± 0.03 | | 7.43 ± 0.06  7.41 ± 0.02^ll^  7.46 ± 0.03 | | 7.43 ± 0.05  7.43 ± 0.03^ll^  7.47 ± 0.02 |
| PaO_2_/FiO_2_  (mmHg) | NE  Ang II  Sham | 320 ± 48  321 ± 31  345 ± 27 | 262 ± 35  254 ± 34 | 274 ± 81  287 ± 51  316 ± 29 | | 260 ± 43  269 ± 78  340 ± 55 | | 263 ± 33  292 ± 103  328 ± 47 |
| PaCO_2_  (mmHg) | NE  Ang II  Sham | 44 ± 2  47 ± 4  46 ± 3 | 52 ± 6^§^  54 ± 4^##^ | 50 ± 8  57 ± 7^ll^  52 ± 2 | | 51 ± 8^§^  55 ± 3^ll^  51 ± 1 | | 48 ± 5^§^  52 ± 4^ll^  51 ± 1 |
| HCO_3_^-^  (mmol/L) | NE  Ang II  Sham | 33 ± 2  33 ± 1  33 ± 2 | 31 ± 23  32 ± 1 | 33 ± 4  34 ± 2  36 ± 1 | | 33 ± 3  34 ± 2  36 ± 1 | | 31 ± 3^**^  33 ± 2  36 ± 1 |
| Hb  (g/dL) | NE  Ang II  Sham | 8.7 ± 0.7  8.5 ± 0.7  9.1 ± 0.7 | 14 ± 1.3^§^  13.6 ± 0.8^ll^ | 8.3 ± 1.14  8.6 ± 1.1  9.5 ± 0.3 | | 10.1 ± 0.9^§^  9.2 ± 1.3  9.6 ± 0.5 | | 10 ± 1.4^§^  9 ± 1.7  9 ± 0.3 |
| Na^+^  (mmol/L) | NE  Ang II  Sham | 133 ± 3  133 ± 2  133 ± 2 | 133 ± 2  133 ± 2 | 133 ± 1  135 ± 2  132 ± 2 | | 133 ± 2  134 ± 2  132 ± 4 | | 133 ± 2  133 ± 2  132 ± 4 |
| K^+^  (mmol/L) | NE  Ang II  Sham | 3.6 ± 0,2  3.9 ± 0.3  3.5 ± 0.1 | 4.1 ± 0.3  4.3 ± 0.5 | 4.2 ± 0.6  4.5 ± 0.7  3.8 ± 0.1 | | 4.2 ± 0.3^§^  4.5 ± 0.9^ll^  3.7 ± 0.4 | | 4.6 ± 0.4^§^  4.6 ± 0.9^ll^  3.8 ± 0.2 |
| Cl^-^  (mmol/L) | NE  Ang II  Sham | 101 ± 2  102 ± 2  100 ± 1 | 100 ± 1  100 ± 2 | 100 ± 3  100 ± 2  98 ± 1 | | 99 ± 1  99 ± 2  100 ± 3 | | 100 ± 2  100 ± 2  99 ± 2 |
| Ca^2+^  (mmol/L) | NE  Ang II  Sham | 1.28 ± 0.1  1.32 ± 0.1  1.33 ± 0.1 | 1.36 ± 0.1  1.28 ± 0.1 | 1.20 ± 0.1  1.14 ± 0.1^ll^  1.24 ± 0.1 | | 1.19 ± 0.1^§^  1.17 ± 0.1^ll^  1.18 ± 0.1** | | 1.14 ± 0.1^§^  1.14 ± 0.1^ll^  1.18 ± 0.** |

**Table S6:** Biological variables. Values are expressed as mean ± SD. *p-value between NE and Ang II groups. ^†^p-value < 0.05 between NE and Sham groups. ^‡^p-value < 0.05 between Ang II and Sham groups. p-value < 0.05 compared to baseline for NE (^§^), Ang II (^ll^) and Sham (**) groups. ASAT: Aspartate aminotransferase; ALAT Alanine aminotransferase; LDH: Lactate dehydrogenase.

| VARIABLES  Mean + SD |  | Baseline | | Fluids | | Vasopressor 1 | Vasopressor 2 |  |
| --- | --- | --- | --- | --- | --- | --- | --- | --- |
| BUN  (mg/dL) | NE  Ang II  Sham | 8 ± 2.6  2.6 ± 3.4  7 ± 1.5 | 13.3±3.2^§^  11.7 ± 4.3^ll^  10.4 ± 1.5 | | 15.1 ± 2.9^§^  13.1 ± 4.8^ll^  12.6 ± 2** | | 16 ± 3.7^§^  14.5 ± 4^ll^  14.3 ± 2.3** | |
| ASAT  (UI/L) | NE  Ang II  Sham | 36 ± 13  32 ± 7  0.9 ± 0.19 | 42 ± 20  44 ± 23  0.9 ± 0.2 | | 69 ± 36  129 ± 110^ll^  0.9 ± 0.2 | | 108 ± 66  169 ± 162^ll^  0.9 ± 0.2 | |
| ALAT  (UI/L) | NE  Ang II  Sham | 43 ± 7  43 ± 12  47 ± 6 | 27 ± 6^§,†^  26 ± 6^ll^  45 ± 6 | | 23 ± 3^§,†^  27 ± 15^ll^  48 ± 9 | | 28 ± 9^§,†^  34 ± 24^ll^  50 ± 11 | |
| LDH  (UI/L) | NE  Ang II  Sham | 578 ± 78  583 ± 79  664 ± 86 | 529 ± 169  543 ± 161  771 ± 87 | | 660 ± 226  974 ± 950  836 ± 152 | | 935 ± 609  1151 ± 1352  817 ± 70 | |
| Total protein  (g/L) | NE  Ang II  Sham | 50 ± 3  51 ± 5  49 ± 3 | 37 ± 4^†,§^  36 ± 4^‡,ll^  47 ± 1 | | 29 ± 2^†,§^  29 ± 4^‡,ll^  46 ± 2 | | 26 ± 5^†,§^  28 ± 5^‡,ll^  45 ± 1 | |

**Figure S2:**

1. Left ventricular mRNA expression of molecules implicated in Ca^2+^ handling and contractile apparatus [ATPase sarcoplasmic/endoplasmic reticulum Ca^2+^ transporting 2 (SERCA2A) and phospholamban (PLB)]
2. LV mRNA expression of the ratio Bax/Bcl2 in norepinephrine (black bars) and angiotensin II groups. *p-value<0.05.
3. Cardiac apoptotic rate: ratio of apoptotic nuclei (TUNEL-positive or brown nuclei) to total nuclei (brown+blue nuclei) (x100 to be expressed as a percentage).
4. mRNA expression of cell adhesion molecules (ICAM1, 2 and VCAM1) and eNOS, iNOS, nNOS in norepinephrine and angiotensin II groups compared to sham group.


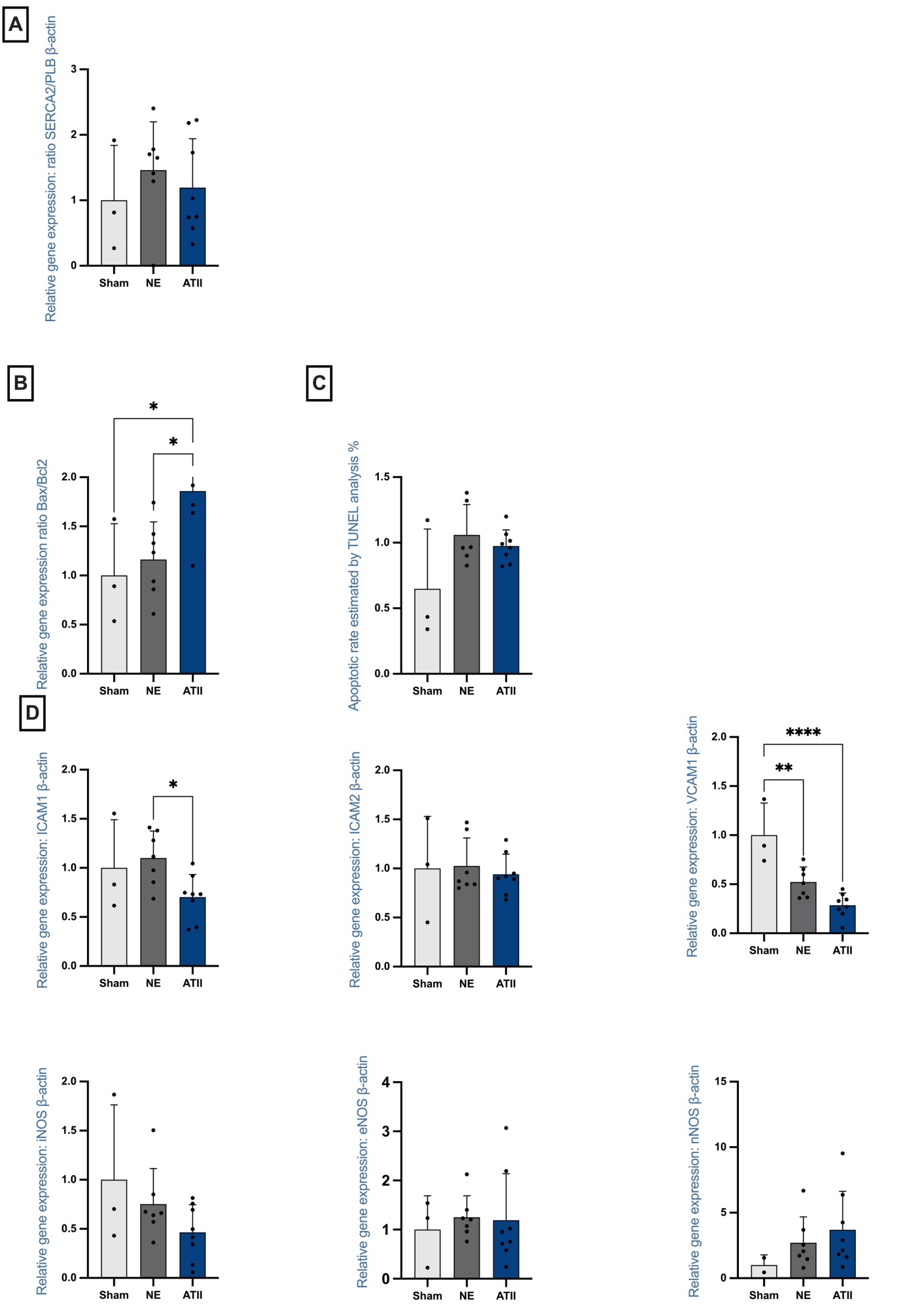


**Figure S3:**

Fold Changes expressed in % between baseline and vasopressor 2 time points

**Ang II NE**

1. Monnet X, Ghaleh B, Lucats L, Colin P, Zini R, Hittinger L, Berdeaux A. Phenotypic adaptation of the late preconditioned heart: Myocardial oxygen consumption is reduced. *Cardiovasc Res*. 2006;70:391–398.

2. Burkhoff D, Mirsky I, Suga H. Assessment of systolic and diastolic ventricular properties via pressure-volume analysis: a guide for clinical, translational, and basic researchers. *Am J Physiol-heart C*. 2005;289:H501–H512.

3. Bastos MB, Burkhoff D, Maly J, Daemen J, Uil CA den, Ameloot K, Lenzen M, Mahfoud F, Zijlstra F, Schreuder JJ, Mieghem NMV. Invasive left ventricle pressure–volume analysis: overview and practical clinical implications. *European Heart Journal*. 2019;41.

4. Vasques-Nóvoa F, Laundos TL, Cerqueira RJ, Quina-Rodrigues C, Soares-dos-Reis R, Baganha F, Ribeiro S, Mendonça L, Gonçalves F, Reguenga C, Verhesen W, Carneiro F, Paiva JA, Schroen B, Castro-Chaves P, Pinto-do-Ó P, Nascimento DS, Heymans S, Leite-Moreira AF, Roncon-Albuquerque R. MicroRNA-155 Amplifies Nitric Oxide&sol;cGMP Signaling and Impairs Vascular Angiotensin II Reactivity in Septic Shock. *Crit Care Med*. 2018;46:e945–e954.
