# Supplementary material for: Myocardial effects of angiotensin II compared to norepinephrine in an animal model of septic shock
Source: Crit Care. 2022 Sep 18;26:281. doi: 10.1186/s13054-022-04161-3 (PMC9482744; doi:10.1186/s13054-022-04161-3)

Figure 3

Uncropped gels of left ventricular phospho-STAT3 and STAT3

STAT3 (Cell Signaling, #9139/ IRDye 680)

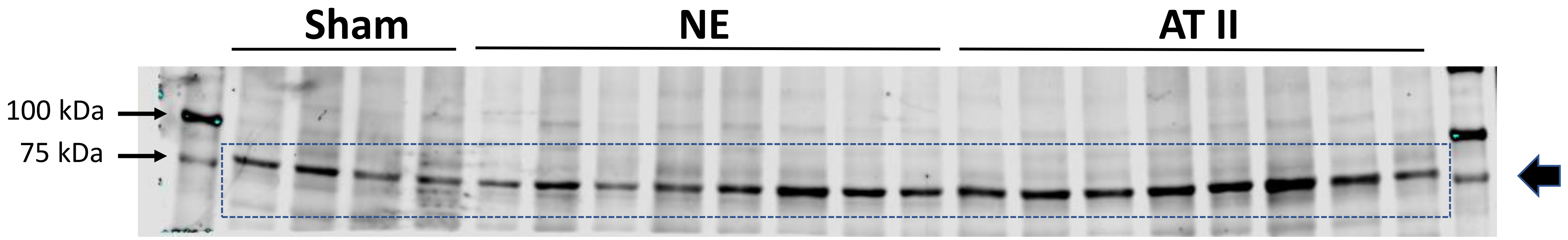

Phospho-STAT3 (Tyr705) (Cell Signaling, #9145/ IRDye 800)

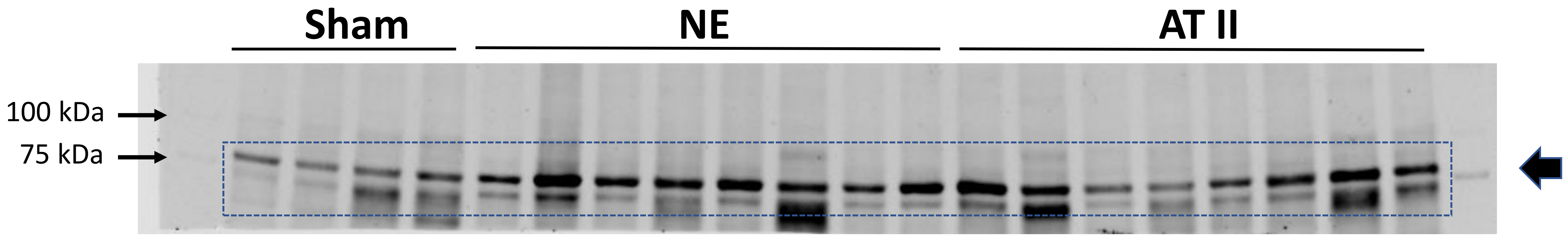

Figure 4

Uncropped gels of alpha-1 adrenoreceptor

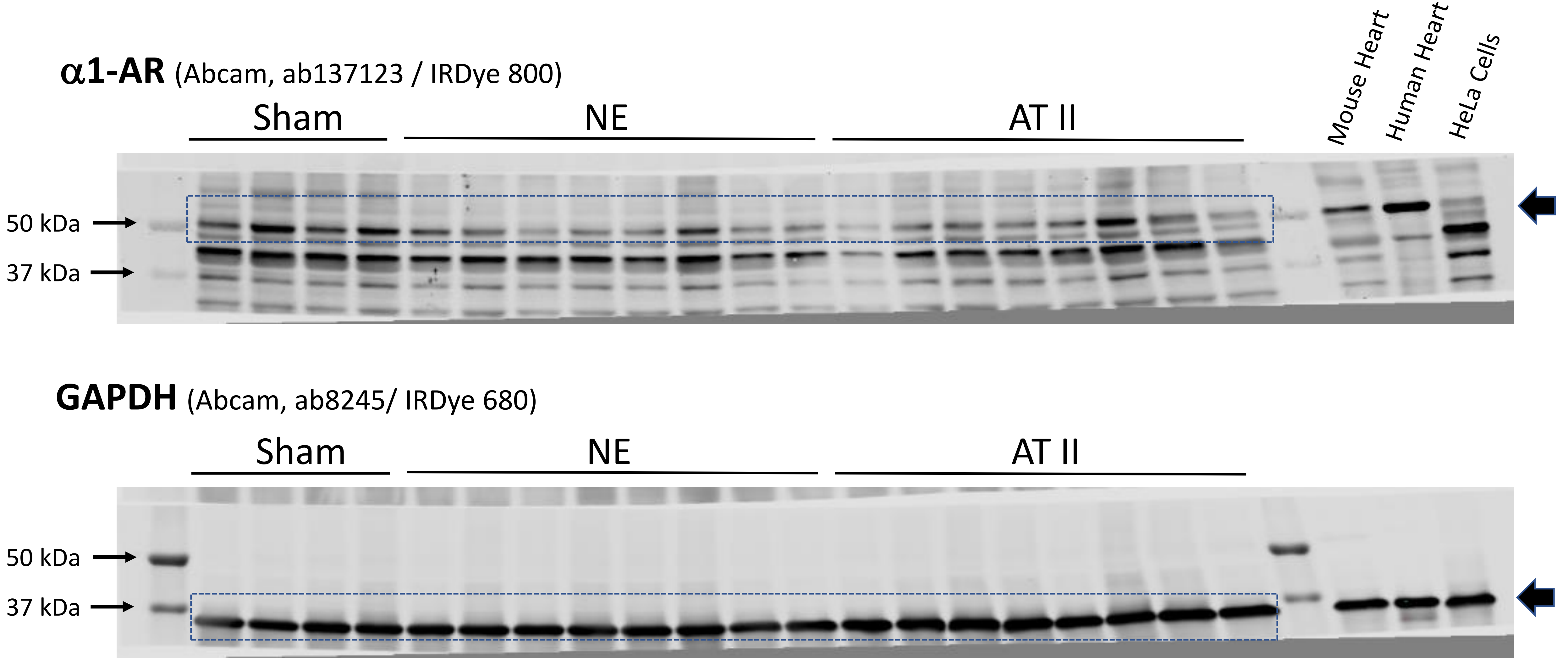

Figure 4

Uncropped gels of left ventricular beta 1-adrenoreceptor

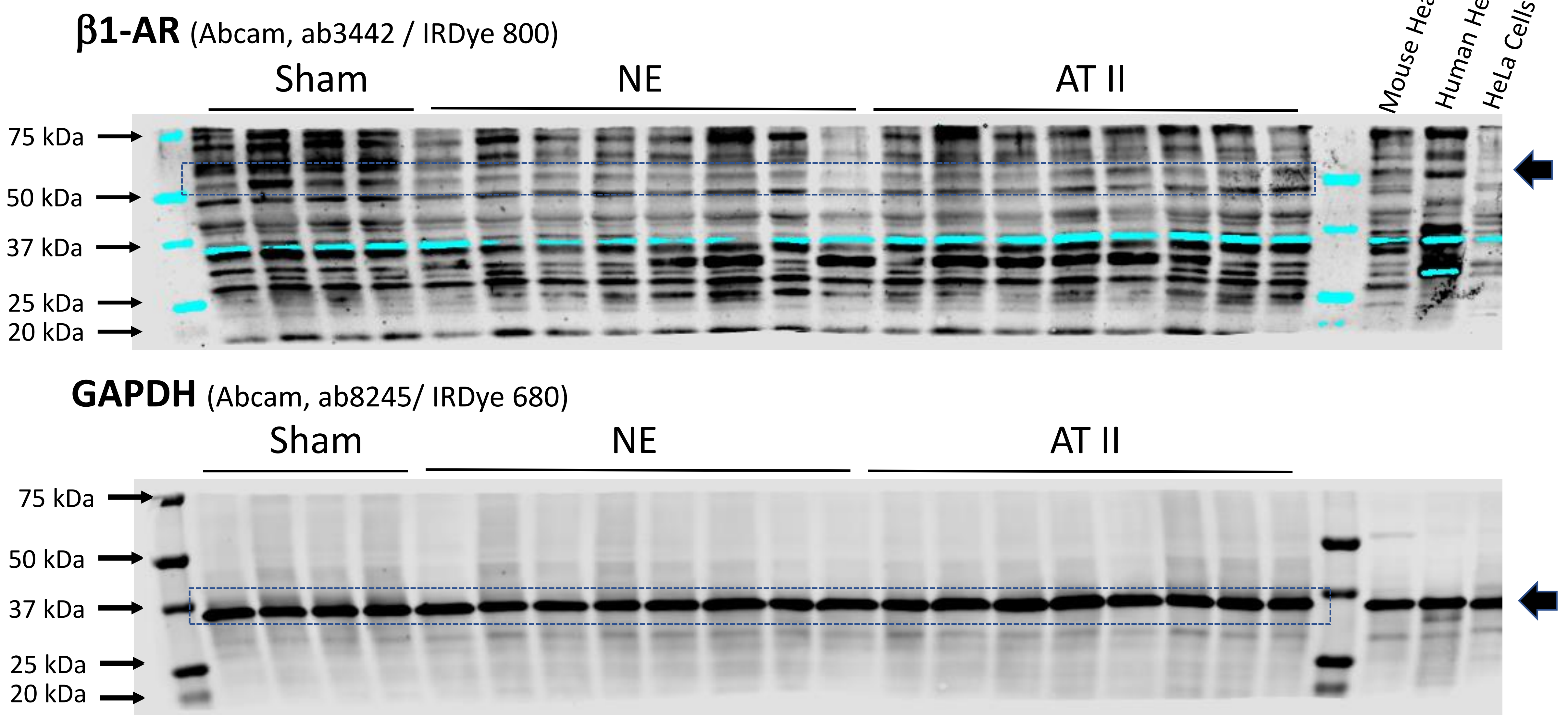

Figure 4

Uncropped gels of left ventricular angiotensin-1 receptor

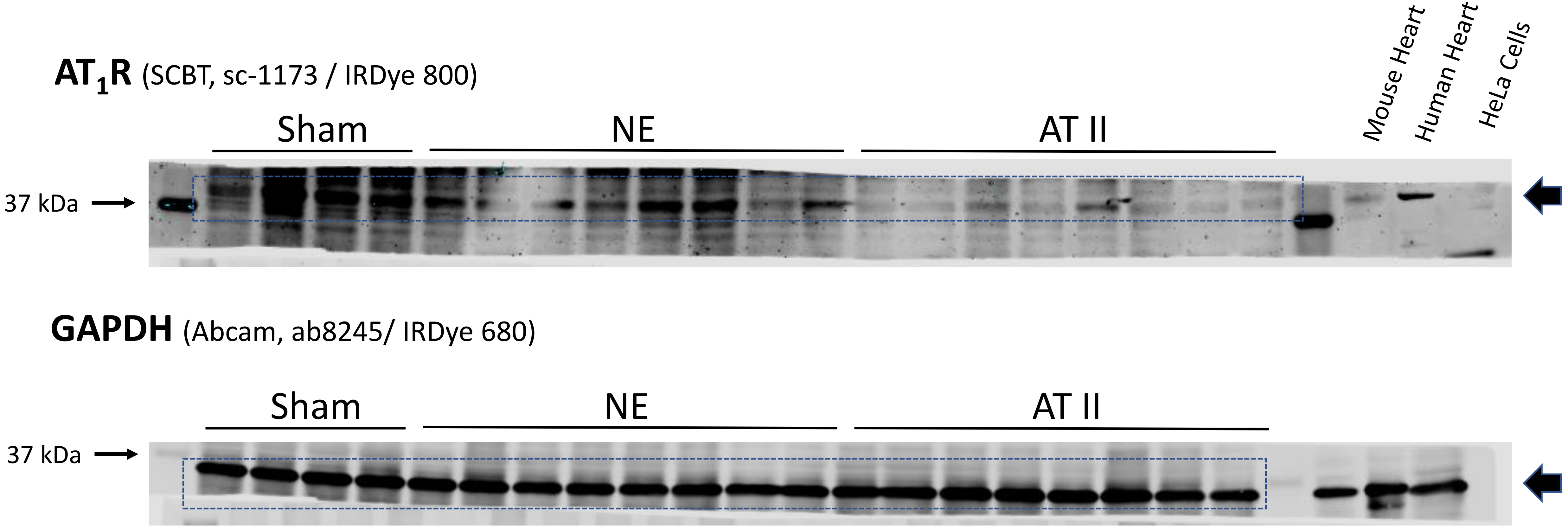

Figure 4

Uncropped gels of left ventricular AGTRAP

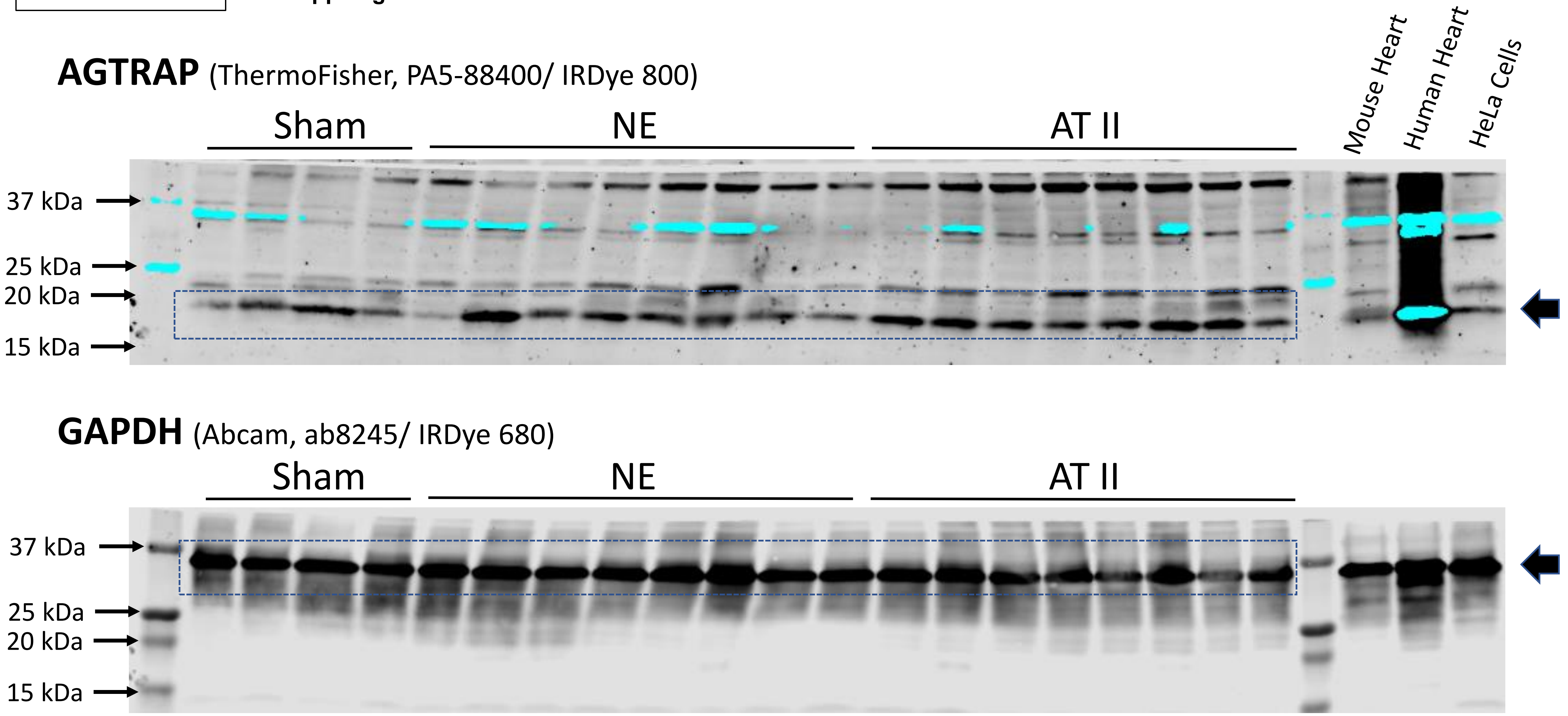

Figure 4

Uncropped gels of left ventricular angiotensin 2 receptor

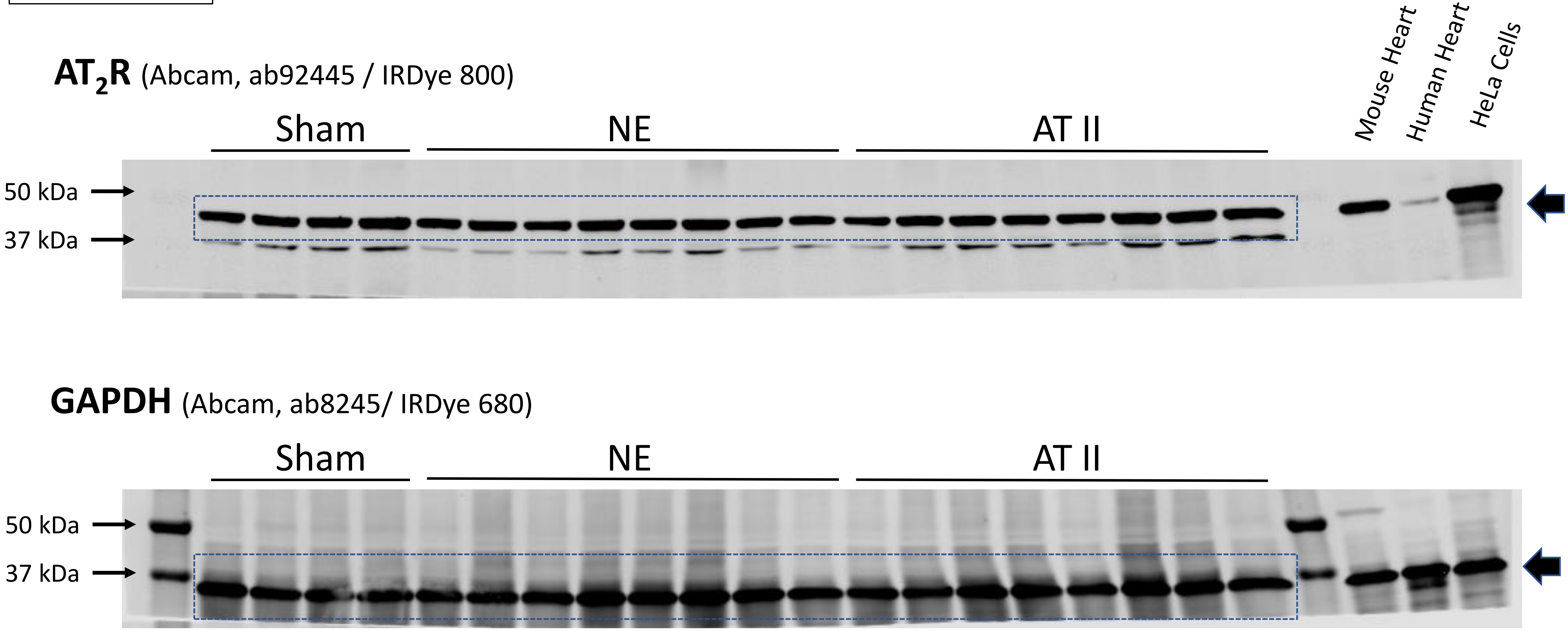

Supplement: Supplementary file 2 — Additional file 2. Uncropped gels. [file 13054_2022_4161_MOESM2_ESM.pdf]
